# Drug-Eluting Fibers for HIV-1 Inhibition and Contraception

**DOI:** 10.1371/journal.pone.0049792

**Published:** 2012-11-28

**Authors:** Cameron Ball, Emily Krogstad, Thanyanan Chaowanachan, Kim A. Woodrow

**Affiliations:** Department of Bioengineering, University of Washington, Seattle, Washington, United States of America; Burnet Institute, Australia

## Abstract

Multipurpose prevention technologies (MPTs) that simultaneously prevent sexually transmitted infections (STIs) and unintended pregnancy are a global health priority. Combining chemical and physical barriers offers the greatest potential to design effective MPTs, but integrating both functional modalities into a single device has been challenging. Here we show that drug-eluting fiber meshes designed for topical drug delivery can function as a combination chemical and physical barrier MPT. Using FDA-approved polymers, we fabricated nanofiber meshes with tunable fiber size and controlled degradation kinetics that facilitate simultaneous release of multiple agents against HIV-1, HSV-2, and sperm. We observed that drug-loaded meshes inhibited HIV-1 infection *in vitro* and physically obstructed sperm penetration. Furthermore, we report on a previously unknown activity of glycerol monolaurate (GML) to potently inhibit sperm motility and viability. The application of drug-eluting nanofibers for HIV-1 prevention and sperm inhibition may serve as an innovative platform technology for drug delivery to the lower female reproductive tract.

## Introduction

HIV-1 infections and unintended pregnancies are inextricably linked by unprotected sex and represent a major health burden for women worldwide [1]. Access to MPTs combining safe, effective, and easily reversible options for contraception are essential for women who are also at risk for STIs including HIV-1. To date, no single product exists that women can use discreetly for simultaneous and effective prevention of STIs and pregnancy [2]. Women would also benefit from more options for chemical contraceptives that are nonhormonal [3], [4]. In particular, an alternative to nonoxynol-9 (N-9) that is safe and non-toxic, does not alter the vaginal microflora, and does not enhance the risk for HIV-1 infection would have a major impact on women's sexual and reproductive health [5].

The materials chemistry and process of fabricating MPTs have a significant influence on their design and function. Existing MPTs are classified by their function as physical, chemical, or combined physical/chemical barriers to prevent STIs and unintended pregnancy [6], [7]. Chemical barrier methods are the front-line approach being evaluated in clinical trials for multipurpose protection, and include dosage forms such as gels, films, tablets, and intravaginal rings (IVRs) [8], [9]. Gels containing tenofovir [10], UC781, dapivirine [11], and MIV-150 [12] are among the many anti-HIV-1 microbicides under development. Vaginal films have long existed for the prevention of pregnancy (Vaginal Contraceptive Film® by Apothecus) as well as the treatment of fungal and bacterial infections [9]. Recently, researchers have begun to formulate vaginal films and tablets that can provide quick release of antiviral compounds as an alternative dosage form to microbicide gels. For example, Akil et al. developed thin vaginal films that release 0.6 mg of dapivirine within 10 minutes following hydration [13]. IVRs, which are designed to provide sustained release of a combination of agents over several weeks or months, are the leading technologies being evaluated currently for multipurpose prevention [14], [15]. However, the paucity of materials and the fabrication process for IVRs imposes constraints on their physical design (size, geometry, mechanical properties) and the pharmaceutical agents that are suitable for delivery [8]. Greater flexibility in the choice of polymers as well as the fabrication process could lead to innovative new dosage forms for a larger number of pharmaceutical agents.

Emerging technologies that integrate physical and chemical barriers provide the greatest potential for new MPT designs. To be effective, these technologies must address five fundamental design requirements: 1) deliver multiple drugs with differing physicochemical or pharmacokinetic properties from a single device, 2) provide extensive coverage of mucosal tissue, 3) be female-controlled and discreet, 4) deliver contraception that is fully reversible, and 5) be inexpensive so as to reach the most relevant populations. Based on these requirements, we have developed an innovative dosage form for vaginal drug delivery using drug-eluting nanofibers. Electrospinning is an elegant method to deliver combination drug therapies because polymers can be selected based on their drug compatibility as well as their degradation or dissolution rates [16]. In addition, controlling processing parameters (applied voltage, polymer flow rate, capillary-collector distance), nozzle configuration (single, multijet, coaxial), and choice of materials (non-degradable, biodegradable, water-soluble) allows greater versatility and flexibility to design topical prevention strategies [17].

To demonstrate the versatility of drug-eluting fibers for topical delivery to the vaginal mucosa in applications for multipurpose prevention, we generated nanofiber meshes that elute small molecule agents that target HIV-1, HSV-2, and sperm function. We demonstrated that these drug-eluting fibers deliver antiviral drugs with diverse physicochemical properties and mechanisms of action. Fibers loaded with antiretroviral drugs showed potent inhibition of HIV-1 infection *in vitro*. To address the need for contraception in a multipurpose prevention strategy, we screened multiple non-hormonal chemical contraceptive alternatives to N-9 and discovered an unreported function of glycerol monolaurate (GML) to inhibit sperm motility and viability in a dose-dependent manner. We also showed that fiber meshes acted as a physical barrier to sperm penetration despite their porosity. The fiber meshes were readily electrospun in a geometry designed to provide physical coverage of both the vaginal epithelium and cervix. The functional combination offered by our drug-eluting nanofibers cannot be accomplished with any single technology currently in the development pipeline.

## Results

### Electrospun fibers incorporate antiviral compounds with high drug loading

**Figure 1:**
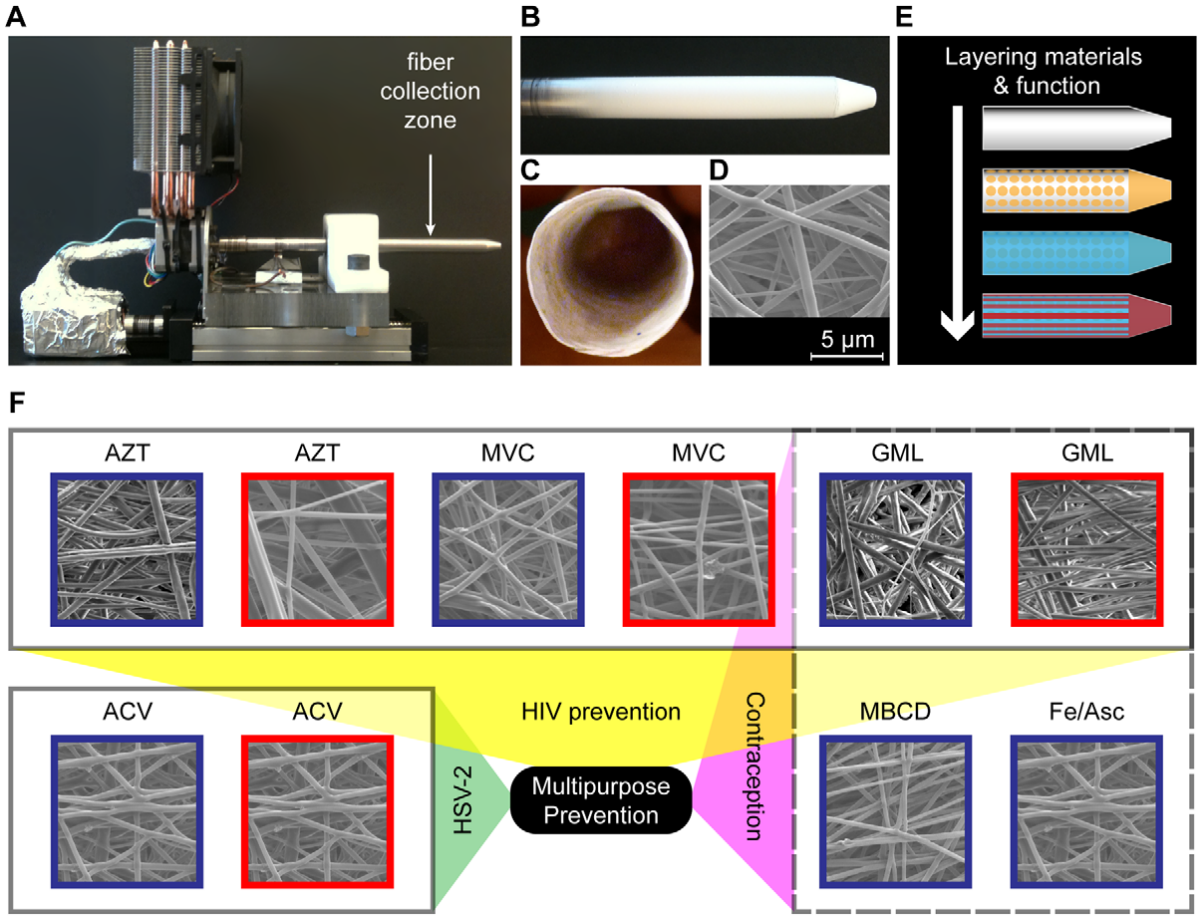
Electrospun fibers incorporate drugs for multipurpose prevention. (**a**) Two-axis mandrel electrospinning rig for fiber collection. (**b**) Controlled fiber deposition along a grounded aluminum collector produces a geometry that may be suitable for vaginal drug delivery. (**c**) Mesh abstracted from mandrel has a hollow interior. (**d**) Fiber meshes have porous microstructure. (**e**) Combining fiber meshes produces a multifunctional material. (**f**) Diverse agents with action against HIV, HSV-2, or sperm are incorporated into blends of PLLA and PEO. PLLA/PEO (30∶70, blue) and PLLA/PEO (70∶30, red); AZT  = 1 wt% 3′-azido-3′-deoxythymidine, MVC  = 1 wt% maraviroc, ACV  = 1 wt% acycloguanosine, GML  = 10 wt% glycerol monolaurate, MBCD  = 10 wt% methyl-β-cyclodextrin, Fe/Asc  = 10 wt% iron (II) D-gluconate with 10 wt% ascorbic acid.

We electrospun fibers from mixtures of hydrophilic polyethylene oxide (PEO) and hydrophobic poly-L-lactic acid (PLLA), two polymers with proven biocompatibility and FDA approval for use in medical implants [18], [19]. We hypothesized that fibers with partially hydrophilic and partially hydrophobic composition would enable encapsulation of agents with high and low aqueous solubility, respectively. Previous studies with PLLA-PEO polymer blends found that blends act as a single material with averaged properties when mixed at a ratio up to 30∶70 or 70∶30 (wt/wt), but act as a composite of two materials with discrete properties when mixed at ratios approaching 50∶50 (wt/wt) [20]. To fabricate homogeneous fibers with uniformly blended hydrophilicity/hydrophobicity, we electrospun 70∶30 (wt/wt) PLLA/PEO and 30∶70 (wt/wt) PLLA/PEO meshes. We also electrospun pure PLLA and pure PEO meshes. Polymer concentration in the electrospinning solution had a significant impact on the resulting fiber diameters between formulations with the same PLLA/PEO ratio and solvent choice, as assessed by ANOVA (P<0.0001) (**Fig.** **S1**). Electrospinning parameters were modified to yield fibers with reproducible size and high polymer recovery (**Fig.** **S1** **–** **2**), and two blends were identified for further study: 70∶30 PLLA/PEO in 1∶1 chloroform/2,2,2-trifluoroethanol and 30∶70 PLLA/PEO in 3∶1 chloroform/2,2,2-trifluoroethanol. These compositions produced fiber diameters of 200–700 nm and polymer recovery of >50% (**Fig.** **S1** **–** **2**). The fibers were collected on a mandrel designed in the geometry of a tampon applicator (**Fig.** **1a–b**) and resulted in fiber meshes in the shape of a hollow tube (**Fig.** **1c**), which we were able to incorporate into a standard tampon applicator (**Fig. S3**). By controlling the axial deposition of the fibers near the apex of the collector, we could also form a thick barrier mesh (∼2–3 mm thick) that is continuous with a thinner inner mesh (down to ∼10 μm thick).

We selected several model compounds to demonstrate the versatility of electrospun fibers to deliver agents with differing solubility and mechanisms of action against either HIV-1 or HSV-2 (**Fig. 1f**). We electrospun fibers containing either 1% (wt/wt) maraviroc (MVC), which inhibits CCR5-mediated HIV fusion, 1% (wt/wt) 3′-azido-3′-deoxythymidine (AZT), which inhibits viral reverse transcriptase, or 1% (wt/wt) acyclovir (acycloguanosine), which has antiviral activity against HSV-2. Collectively, these compounds vary in aqueous solubility (0.01–50 g L^−1^) and span a wide range of log P values (−1 to 4). We assessed drug loading of MVC or AZT-loaded fiber meshes stored at room temperature (19–22°C) for at least five months by dissolving them in acetonitrile and measuring drug content with HPLC. MVC and AZT were incorporated successfully into fibers at >95% drug encapsulation efficiency for both PLLA/PEO blend compositions. ARVs eluted from the polymer fibers were identical to the unformulated drugs as measured by UV-HPLC (**Fig. S4**), suggesting that the compounds are stable during electrospinning and during shelf storage for at least five months. Fiber meshes retained the same white color and soft, flexible texture over five months. While electrospinning did not compromise drug integrity, we found that drug incorporation into polymer fibers could influence fiber size, fiber alignment, and polymer recovery (**Fig. S5–S8**).

### Drug-loaded fibers erode and release agents to potently inhibit HIV-1 activity in vitro

**Figure 2:**
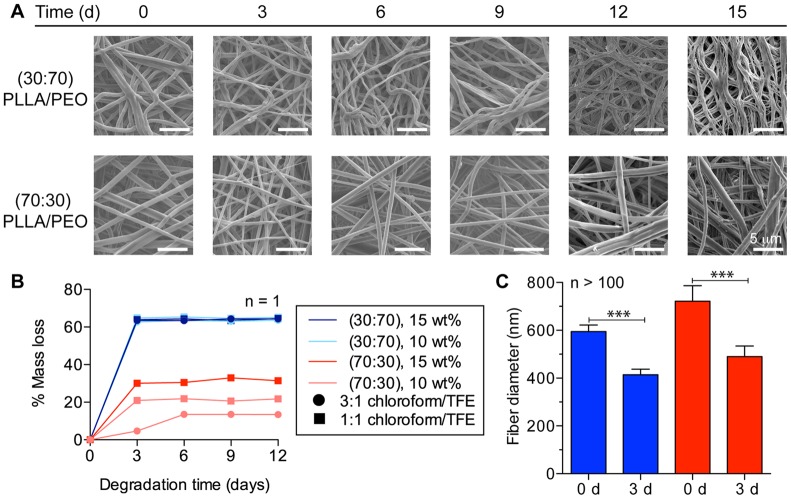
Fiber composition influences degradation properties. (**a**) SEM micrographs show that fiber and mesh morphology changes markedly over 15 d in VFS. (**b**) Mass loss of fibers over time is controlled by PEO content in fibers. (**c**) Fiber diameters, displayed as geometric mean with 95% confidence interval, and decrease significantly over three days of degradation in VFS (p<0.0001 for 30∶70 and 70∶30 PLLA/PEO fibers). 30∶70 PLLA/PEO (blue) and 70∶30 PLLA/PEO (red) for (**b**) and (**c**).

Sustained drug release over weeks to months has potential for greater adherence whereas burst release of active agents may be desirable for pericoital prevention [2]. Since degradation of polymeric delivery systems can influence drug release properties, we fabricated fibers with varying degradation rates by modulating the hydrophilic and hydrophobic content of the fibers. We monitored fiber degradation in VFS over two weeks by recording mass loss and imaging with SEM (**Fig.** **2**). Fiber meshes with greater hydrophilic content showed the most pronounced change in individual fiber and overall mesh morphology (**Fig.** **2a**). We observed that fibers decreased in diameter within hours to days, and then appeared to fuse into large fiber bundles. These observations were confirmed by measuring a 30% (95% CI  = 25% to 35%, n = 117) reduction in 30∶70 PLLA/PEO and a 32% (95% CI  = 23% to 40%, n = 103) reduction in 70∶30 PLLA/PEO fiber diameters after 3 days and over 30% mass loss of the meshes within one week (**Fig.** **2b, c**). The percent mass loss corresponded with the percent PEO composition in the fibers (**Fig.** **2b**). Pure PLLA fibers showed no mass loss after a one-hour incubation in VFS, whereas pure PEO fibers dissolved in less than 10 minutes upon contact with water.

**Figure 3:**
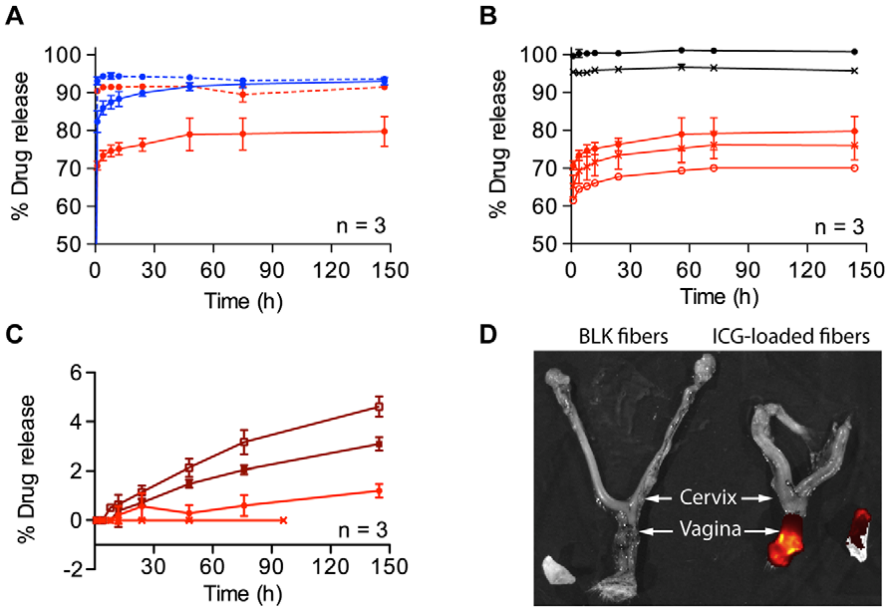
Fibers release active antiretroviral agents. (**a**) Cumulative drug release in VFS was measured for 30∶70 PLLA/PEO (blue) and 70∶30 PLLA/PEO (red). AZT (dashed line) and MVC (solid line) showed rapid burst release from blended fibers within 1 h. (**b**) Varying fiber diameter resulted in MVC burst release from PCL fibers (black) and 70∶30 PLLA/PEO fibers (red). PCL meshes with two fiber diameters ( •  = 370 nm and ^⊥^  = 1.3 μm) and 70∶30 PLLA/PEO fibers with three fiber diameters ( •  = 560 nm, ○  = 1.5 μm, ^⊥^  = 3.4 μm) were tested. (**c**) Sustained release of MVC is achieved from PDLLA/PLLA blends and from 99∶1 PLLA/PEO, but not from PLLA fibers. 50∶50 PDLLA/PLLA (□), 25∶75 PDLLA/PLLA (▪), 99∶1 PLLA/PEO (•), and 100% PLLA (^⊥^). (**d**) Insertion of fibers into mouse vagina and subsequent fluorescent imaging reveal release of dye within 30 minutes for ICG-loaded fibers (right) compared with blank fibers (left). Fiber meshes are shown next to excised reproductive tracts.

To investigate if drug release kinetics recapitulated polymer degradation kinetics, we monitored drug release from meshes incubated in VFS (**Fig.** **3**). We observed that AZT and MVC burst released from fibers within 1 h, but that the drug release profiles differed based on PLLA and PEO content of the fibers. For example, fibers with greater hydrophilic content (30∶70 PLLA/PEO) released 2.1% more AZT (95% CI  = 0.68% to 3.5%, n = 3) and 13% more MVC (95% CI  = 6.8% to 20%, n = 3) over 6 d than corresponding meshes with greater hydrophobic content (70∶30 PLLA/PEO) (**Fig.** **3a**). In 6 d, 70∶30 PLLA/PEO fibers released 92±0.75% of encapsulated AZT and 80±3.9% of MVC into VFS (n = 3). The 30∶70 PLLA/PEO fibers released 94±0.48% of encapsulated AZT and 93±0.98% of MVC into VFS (n = 3). We did not detect MVC release from pure PLLA fibers over 96 h in VFS (**Fig.** **3c**). These results suggest that controlling polymer-drug interactions and the rate of polymer swelling and dissolution may alter the release kinetics of different active agents. Given the burst release of AZT and the aqueous solubility of indocyanine green (ICG) dye, we chose to electrospin ICG-loaded fibers to investigate the extent of fiber coverage and agent release in mice. After inserting 30∶70 PLLA/PEO fibers loaded with 1% (wt/wt) ICG into mice, we observed that dye completely coated the vaginal tract after 30 minutes (**Fig.** **3d**). This study provides initial evidence that fibers are able to sufficiently hydrate and release agents to coat the vaginal mucosa *in vivo*.

We tested multiple strategies to obtain sustained release of MVC from fibers by increasing fiber diameter, reducing hydrophilic polymer content, and modulating PLA crystallinity. MVC was chosen to establish proof of principle for controlled release because it was less hydrophilic than AZT. First, we tested the hypothesis that increasing fiber diameter would slow release of MVC from 70∶30 PLLA/PEO fibers. We increased fiber diameters by raising the polymer concentration in 70∶30 PLLA/PEO solutions (**Fig. S9,10**). Despite three- and six-fold increases in fiber diameter, all 70∶30 PLLA/PEO fibers burst released MVC within one hour in VFS (**Fig.** **3b**). Comparison of mean MVC release from variably sized 70∶30 PLLA/PEO fibers with ANOVA showed that the fibers released significantly different amounts of MVC based on fiber size (P = 0.0261). The data suggested a trend for larger fiber diameters to release less MVC into VFS over six days, and 3.4 μm diameter fibers released 9.6% less MVC (95% CI  = 1.17% to 18.1%, n = 3) than 560 nm fibers.

Our second strategy was to reduce hydrophilic polymer content by electrospinning fibers from a 99∶1 PLLA/PEO blend containing 1% (wt/wt) MVC. The resulting fibers were smooth, regular, and similarly sized to 70∶30 and 30∶70 PLLA/PEO fibers (**Fig. S10**). When placed into VFS, the fibers showed no burst release of MVC. Rather, the fibers provided sustained release over six days, eluting an average of 1.19% of encapsulated MVC into VFS (95% CI  = 0.51 to1.88%, n = 3) (**Fig.** **3c**).

Thirdly, we encapsulated 1% (wt/wt) MVC into fiber meshes made from polycaprolactone (PCL) or blends of poly-(D, L)-lactic acid (PDLLA) and PLA to investigate the influence of polymer crystallinity on release rate. PCL is a bioabsorbable hydrophobic polymer with a long history of use in electrospinning [21]. PCL has a much lower melting temperature than PLLA, reflecting lower crystallinity and greater molecular flexibility in the polymer strands [22]. We electrospun PCL meshes containing 1% (wt/wt) MVC with two different fiber diameters: 371 nm and 1.3 μm (**Fig. S10**). Upon incubation in VFS, all PCL meshes burst released more than 95% of the encapsulated MVC within 1 hour (**Fig.** **3b**). Larger PCL fibers released 5.04% less MVC over 6 days (95% CI  = 3.92% to 6.17%, n = 3) than small PCL fibers.

We modulated the crystallinity of pure PLA fibers by blending PLLA with varying amounts of lower molecular weight poly(D, L-lactide) (PDLLA). PDLLA is chemically identical to PLLA, but displays key structural differences. In particular, PDLLA is amorphous, and allows for greater penetration of water into PLA meshes [20]. We electrospun 25∶75 and 50∶50 PDLLA/PLLA blends containing 1% MVC. The resulting fibers showed regular morphology with similar fiber size (265±145 nm and 190±159 nm, respectively) to pure PLLA fibers (478 nm±287 nm) (**Fig. S10**). When placed into VFS, these PDLLA/PLLA meshes showed no burst release, and released MVC linearly over six days. The amount of encapsulated MVC released after six days was 3.09±0.27% from 25∶75 PDLLA/PLA fibers and 4.61±0.41% from 50∶50 PDLLA/PLLA fibers. 50∶50 PDLLA/PLLA meshes released significantly more MVC than 25∶75 PDLLA/PLLA meshes (P value  = 0.0059, n = 3). Therefore, modulating the polymer crystallinity by blending PDLLA with PLLA provided small, but sustained, linear release of MVC from electrospun fibers.

**Figure 4:**
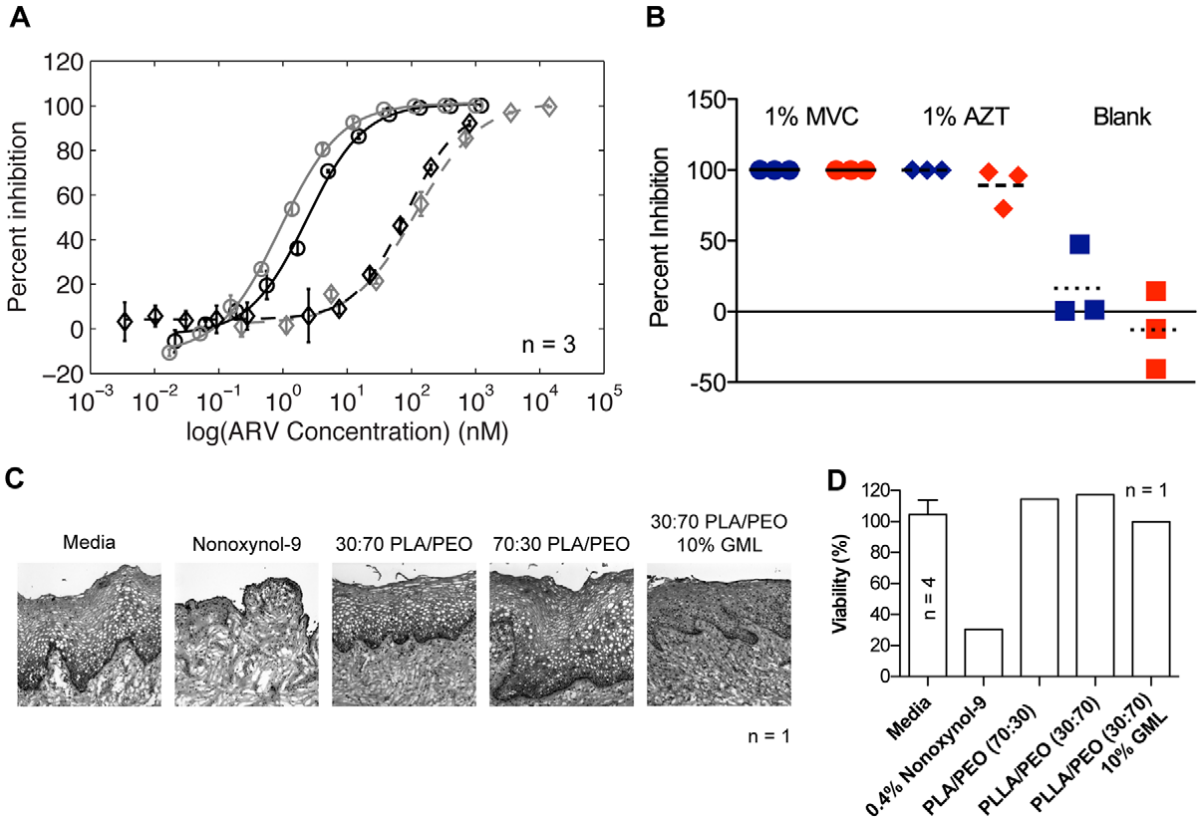
Fiber meshes inhibit HIV *in vitro* and are nontoxic to macaque cervical tissue explants. (**a**) Dose-response assay indicates that AZT and MVC released from fibers have similar potency to unformulated drugs (drug eluates, black and unformulated drug, gray). (**b**) Drug loaded fiber blends (30∶70 PLLA/PEO (blue) and 70∶30 PLLA/PEO (red)), but not blank fiber controls, show equivalent inhibition of HIV infection. (**c**) Histology indicates that 30∶70 PLLA/PEO, 70∶30 PLLA/PEO, and 30∶70 PLLA/PEO fibers with 10% (wt/wt) GML are nontoxic to macaque cervical tissue explants compared to nonoxynol-9 control. (**d**) MTT assay confirms fibers, including those containing 10% (wt/wt) GML, are nontoxic to tissue explants. Note that for media controls n = 4, and for all other groups n = 1.

We evaluated the activity and toxicity of our drug-loaded fibers in several relevant *in vitro* assays. MVC, AZT, and fibers were shown to be nontoxic to TZM-bL cells, and no difference between treated cells and media controls was found (Bonferroni post test, α = 0.05) (**Fig. S11**). We tested the ability of both the drug *eluates* released from 70∶30 PLLA/PEO and 30∶70 PLLA/PEO fibers and the drug-loaded fibers *themselves* to inhibit HIV-1 BaL infection in TZM-bL cells. First, we determined the specific antiviral activity of drug eluates released from the fibers to confirm that the absolute drug activity was not diminished by electrospinning. We measured an IC50 value of 0.90 nM and 2.3 nM for unformulated and eluted MVC, respectively. The IC50 of unformulated and eluted AZT was found to be 120 nM and 84 nM, respectively (**Fig.** **4a**). The order of magnitude agreement between drug IC50 values before and after spinning suggests that the stabilities of MVC and AZT are maintained during electrospinning. Incubating TZM-bL cells with drug-loaded fiber discs significantly inhibited HIV-1 infection compared to blank fibers (P value <0.0001) (**Fig.** **4b**). The polymer composition of the mesh at this dosing did not impact their anti-HIV activity, and we saw equivalent viral inhibition for both drugs using the 30∶70 and 70∶30 PLLA/PEO meshes (Bonferroni post test, α = 0.05). Fiber toxicity was evaluated in an *ex vivo* tissue explant model using macaque cervical tissue. In contrast to tissue treated with N-9, we observed no reduction in tissue viability due to exposure to blank fibers or fibers loaded with 10% (wt/wt) GML as determined using an MTT assay and by histological examination of tissue morphology (**Fig. 4c, d**).

### GML fibers are a chemical and physical barrier against sperm function

To address the need for contraception in a multipurpose prevention strategy, we sought to identify non-hormonal chemical alternatives to N-9. We first evaluated the spermicidal capabilities of ferrous D-gluconate (FeGluc) and ascorbic acid (Asc) to corroborate findings from literature that the metal compound and ascorbic acid cause rapid spermiostasis due to lipid peroxidation of sperm [23]. We also evaluated methyl-β-cyclodextrin (MBCD), which we hypothesized might sequester cholesterol from semen and lead to premature sperm capacitation [24]. FeGluc and MBCD were readily incorporated into electrospun fibers (**Fig.** **1** **, Fig. S5–6, 8**), but these agents were ineffective at inhibiting sperm function as assayed by measuring motility of purified (swim-out) human sperm (Table S1).

**Figure 5:**
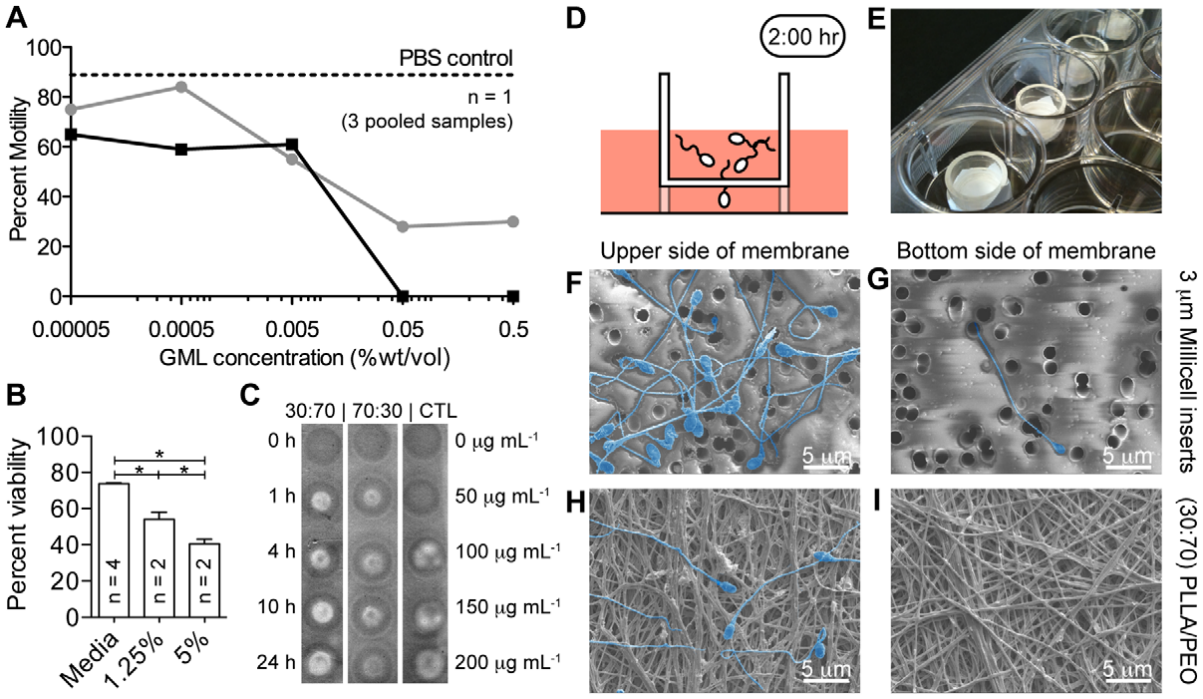
Fiber meshes are a physical and chemical barrier against sperm. (**a**) Motility of human swim-out sperm was completely inhibited within 5 min for 0.05 and 0.5% GML. Data show counts of motile and immotile sperm at 2 min (gray line) and 5 min (black line). Baseline sperm motility (∼89%) was measured at the beginning and end of experiment using a PBS control (dotted line). (**b**) Sperm viability is reduced in whole semen incubated with GML compared with media control. (**c**) GML release from fiber meshes was qualitatively measured using TLC. (**d, e**) A transwell assay was used to test the physical barrier properties of the fiber meshes by replacing Millicell cell culture insert membranes (3 μm pore diameter) with a blank fiber mesh (n = 3). (**f, g**) SEM micrographs of the upper (**f**) and lower (**g**) side of Millicell control membrane. (**h, i**) SEM micrographs of upper (**h**) and lower side (**i**) of fiber mesh show that no sperm penetrate through the fiber mesh.

Based on the amphiphilic properties of glycerol monolaurate (GML) and its reported function to interact with lipid bilayers [25], we were motivated to evaluate GML activity on sperm function. We hypothesized that GML could potentially interact with sperm plasma membranes to reduce sperm viability and motility. Using human swim-out sperm, GML inhibited sperm motility at concentrations of 0.05–0.5% (wt/vol) (**Fig.** **5a**). At these concentrations, complete spermiostasis was measured in <5 min (Video S2, 3). Reduction in motility was also observed at concentrations down to 0.00005% (wt/vol) but did not result in complete spermiostasis during the measurement time (**Fig.** **5a** **, Table S1, and Video S1–S4**). GML also reduced viability of human sperm in whole semen by 33.1% (95% CI  = 24.0% to 42.2%, n = 2) when tested at a 5% (wt/vol) concentration and by 19.6% (95% CI  = 10.7% to 28.9%, n = 2) (**Fig.** **5b**). We also fabricated fibers loaded with 1% or 10% (wt/wt) GML using both PLLA/PEO blends. GML fibers were reproducibly electrospun to achieve polymer recoveries of >70% and fiber diameters between 600–800 nm (**Fig. S5–S8**). Fibers loaded with 10% (wt/wt) GML released ∼100–200 µg mL^−1^ into VFS within 1 h, suggesting that 100% GML released from fibers within 1 hour of incubation with VFS (**Fig.** **5c**).

In addition to encapsulating agents that chemically inhibited sperm function, the fibers served to physically block sperm penetration. We used a transwell assay to measure the ability of sperm to penetrate electrospun meshes in the absence of drugs. The thicknesses of the tissue insert controls and electrospun meshes used as barriers were 30 μm and 150 μm, respectively. We found that motile sperm placed onto electrospun mesh were unable to swim through the fiber meshes despite the presence of numerous pores greater than 3 µm (**Fig.** **5d–e**). In contrast, tissue culture insert controls with 3 μm diameter pores allowed sperm to penetrate into the bottom chamber. Approximately 58,000 sperm mL^−1^ (∼1.7% of sperm) penetrated the commercial membranes in 2 h, whereas no sperm penetrated the fiber meshes (**Table S2**). SEM image analysis confirmed these results, as we observed sperm on the underside of the control membranes but not the electrospun fiber meshes (**Fig.** **5f–i**). To assess the material strength of the electrospun meshes, we performed uniaxial tensile testing on samples of PCL containing 1% MVC that were spun at 5, 50, or 100 μL min^−1^ (n = 2). We found that all materials had a Young's modulus between 25–120 MPa. In addition, materials were able to withstand at least 50% strain before failure. There was a statistically significant difference in Young's moduli between fibers spun at different flow rates, as determined by ANOVA (P = 0.048). A Bonferroni corrected t test was used to compare 5 μL min^−1^ and 50 μL min^−1^ fibers. It was found that fibers spun at a flow rate of 50 μL min^−1^ were on average 68 MPa stiffer than those spun at a flow rate of 5 μL min^−1^ (95% C.I  = 2.8 to 130 MPa stiffer).

## Discussion

We have developed an innovative dosage form for intravaginal drug delivery using drug-eluting fibers fabricated by electrospinning. We show that electrospun fibers deliver agents that inhibit both HIV and sperm *in vitro* in addition to physically preventing sperm penetration. We also report on a novel function of GML to act as a spermicide and potential non-hormonal chemical contraceptive. This finding adds to the characteristics that make GML an attractive candidate for use in topical microbicides for multipurpose prevention [26]. Unlike existing vaginal drug delivery systems, polymer fibers may provide a single dosage form that is readily amenable to encapsulating an array of small molecule hydrophobic and hydrophilic compounds. The diversity and number of polymers that can be electrospun should enable a correspondingly large number of active agents to be encapsulated for sustained delivery [16]. Drug-eluting fibers formulated with a single agent can be assembled into a composite mesh to deliver drug combinations (**Fig.** **1e**). Combined with the ability to control device geometry (**Fig. S3**), we expect that layered chemical function will enable delivery of specific drugs to defined regions within the lower female reproductive tract. The application of drug-eluting fibers for drug delivery to prevent HIV-1 and inhibit sperm function is unprecedented, and should have wide implications for the design of next generation multipurpose prevention technologies.

Topical delivery systems that combine potent and broadly active inhibitors have the greatest likelihood of protecting against the global diversity of STI and HIV variants that are transmitted sexually. The compounds that we incorporated into our fibers have different mechanisms of action against HIV and HSV-2. MVC prevents HIV entry by binding to CCR5 [27] and is already in clinical trials for use as a microbicide (MTN-013/IPM 026). While AZT is not currently a leading candidate for use in topical microbicides, its physicochemical properties are similar to those of tenofovir, which has been used in recent and ongoing clinical trials of microbicide gels [10]. GML, which we report has activity against sperm function, has also been shown to inhibit HIV infection *in vitro* and SIV infection of macaques *in vivo* by inhibiting the production of MIP-3α and other pro-inflammatory cytokines [26]. Finally, we also incorporated ACV into fibers, since HSV-2 infection is of great concern in its own right and in relation to risk of acquiring HIV and other STIs [28]. Together, these four compounds provide a strong proof of principle that electrospun fibers may be a useful platform for vaginal drug delivery and topical prevention of STIs.

For this work, we chose to electrospin fibers from mixtures of PEO and PLLA. We expected PEO to rapidly hydrate and dissolve in vaginal fluid and that PLLA would degrade slowly via hydrolysis at low pH into lactic acid [20], a natural component of vaginal fluid important for maintaining vaginal homeostasis [29]. We found that the magnitude of MVC release from PLLA/PEO blends was highly dependent upon the amount of PEO present. Over six days, 78% of encapsulated MVC was released from 70∶30 PLLA/PEO fibers, compared with 93% of encapsulated MVC from 30∶70 PLLA/PEO fibers. In contrast, approximately 90% of encapsulated AZT was released from both 70∶30 and 30∶70 PLLA/PEO fibers (**Fig. 3**). This suggested that MVC could disperse evenly throughout both PEO and PLLA, while the majority of AZT partitioned into PEO. Small diameter hydrophilic fibers may be an improvement on current film devices that provide coitally dependent protection against STIs and pregnancy. Our data show that substantial amounts of hydrophilic and hydrophobic drugs can be delivered very quickly from nanometer diameter fibers. Meshes made from hydrophilic polymers have previously been found to dissolve and release encapsulated agents more rapidly than films cast from the same materials [30], [31]. Yu et al. showed that 100% of encapsulated ferulic acid dispersed with sodium dodecyl sulfate and sucralose was released within 2 minutes from 250 nm polyvinylpyrrolidone fibers, compared to 20 minutes from equivalent masses of thin films produced by casting. Furthermore, *in vitro* testing of ferulic acid permeation across the sublingual mucosa demonstrated a doubling of permeation rate for the fiber dosage form over the film dosage form [30]. Due to the high surface area-to-volume ratio of electrospun fiber meshes, water may ingress more rapidly into hydrophilic electrospun materials than into cast films. Rapid hydration and a shorter diffusion distance offered by a nanofiber can create a very steep concentration gradient of encapsulated molecules, thus enhancing the rate of mass transport into mucosal tissues [31]. While we did not compare drug release from PLLA/PEO films to fibers, future studies should investigate how nanofibers can enhance the dissolution and mucosal delivery of ARVs.

The ability to hydrate rapidly upon insertion can aid in fast drug release and effective spreading of dissolved materials along the vagina. Material spreading can result in more complete coverage of the mucosal tissue that is vulnerable to infection by HIV and other pathogens [32]. We showed that insertion of indocyanine green loaded 30∶70 PLLA/PEO fibers into the vagina of a mouse resulted in high levels of fluorescence throughout the vaginal tract (**Fig.** **3**). This suggests that fibers can sufficiently hydrate in small volumes of vaginal fluid and release encapsulated agents within 30 minutes *in vivo*. Hydrophilic polymer-based fiber meshes, including those made from PEO, may therefore provide a useful dosage form for pericoital prevention methods.

While rapid release of antivirals and contraceptives is desirable for pericoital prevention, sustained release of agents is desirable for providing extended periods of coverage that may increase user adherence. In contrast to PEO, PLLA is significantly hydrophobic, and fibers with high PLLA content more closely resemble solid depots like IVRs or drug-eluting diaphragms. We hypothesized that polymer fibers with a partially hydrophobic composition would enable encapsulation and sustained release of hydrophobic agents. While our results demonstrated that partially hydrophobic fibers could successfully incorporate MVC and GML (two hydrophobic agents), 70% and 30% PLLA content did not provide sustained release (**Fig.** **3** **, S5–9**), We evaluated multiple strategies for controlling the release of MVC from electrospun fibers by increasing fiber diameter, reducing hydrophilic polymer content, and modulating polymer crystallinity.

Our first strategy for sustaining release of MVC was to electrospin larger diameter fibers (**Fig. S9–10**). While Cui et al. showed that increasing PDLLA fiber diameter from 212 to 551 nm led to slower release of the highly water-soluble compound acetaminophen [33], we observed MVC release from 70∶30 PLLA/PEO fiber meshes was not altered by increasing fiber diameter six fold from 560 nm to 3.4 μm. If the release of MVC from 70∶30 PLLA/PEO fibers were diffusion controlled, an increase in diameter by a factor of six should have decreased release rates by a factor of approximately 36. While we did not observe significant slowing of drug release, we did find that increasing fiber size resulted in a 9.64% (95% CI  = 1.17% to 18.1%) reduction in the amount of drug released after 6 d (**Fig.** **3b**).

We hypothesized that reducing the hydrophilic content in PLLA/PEO blends could mediate sustained release of MVC by reducing the amount of PEO at the surface of the fibers. Our release data from 70∶30 and 30∶70 PLLA/PEO fibers containing 1% (wt/wt) MVC showed that the extent of MVC release was related to PEO content. In fact, we did not detect any MVC release from 100% PLLA fibers in VFS. We electrospun 99∶1 PLLA/PEO fibers to investigate if much smaller amounts of PEO could still allow for hydration of the mesh and release of MVC but prevent burst release. In fact, we observed that fibers composed of 99∶1 PLLA/PEO gave linear release of 1.19±0.28% of encapsulated MVC over six days. This amount of MVC corresponded to an average concentration of 360±120 nM (n = 3) MVC, two orders of magnitude greater than MVC's IC50 *in vitro*. Nevertheless, much of the MVC remained trapped within the PLLA, highlighting that the PLLA/PEO blends that we tested are not ideal for sustained release applications for MVC. However, these results demonstrated proof of principle that controlling the relative amounts of polymers in blended fibers could modify not only the magnitude of release, but also the kinetics of release. The addition of porogens, acid catalysts, or glycolic acid groups in future formulations may enhance water penetration and polyester degradation, thereby improving the magnitude of drug release.

It is unlikely that the hydrophobic nature of PLLA alone was responsible for the lack of MVC release from PLLA. Forbes et al. documented release of MVC from a silicone based elastomer gel both into VFS and into the vaginal fluid of rhesus macaques, providing evidence that MVC can release from hydrophobic formulations [34]. Indeed, we found that MVC burst released from PCL meshes, which are also hydrophobic (**Fig.** **3b**). We hypothesized that the semi-crystalline structure of PLLA is responsible for preventing MVC release from the hydrophobic portions of blended fibers by occluding water penetration. To test this hypothesis, we modulated the crystallinity of PLA fibers by blending PDLLA with PLLA and looked for sustained release of MVC in VFS over six days (**Fig.** **3b** **, S9–10**). PDLLA is comprised of a racemic mixture of the D- and L-stereoisomers of lactic acid and has an amorphous microstructure that allows increased water entry and accelerated polymer degradation compared to PLLA [20]. We found that electrospinning 25∶75 and 50∶50 blends of PDLLA and PLLA allowed the ingress of water and the subsequent linear, sustained release of MVC over six days. 25∶75 and 50∶50 PDLLA/PLLA fibers had similar sizes; 25∶75 fibers were around 20% larger than 50∶50 fibers (**Fig. S10**). The rate of MVC release from PDLLA/PLLA fibers increased with the PDLLA content, but remained small. Cui et al. achieved sustained release of acetaminophen from electrospun PDLLA fibers over six days and found that >70% of encapsulated drug could be released [33]. We found that 50∶50 PDLLA/PLLA fibers only released 4.61±0.41% of MVC into VFS over 6 d. Our results may differ from those of Cui et al. because of MVC's lower aqueous solubility, release media with lower pH (4.2 compared with 7.4), and different PLA isomer composition. Despite the low levels of drug release, our results provide proof of principle that electrospun fibers can sustain release of ARVs over multiple days. It is likely that encapsulating MVC in pure PDLLA would result in greater release of MVC due to reduced crystallinity and accelerated polymer degradation.

In addition to releasing ARVs, it is also critical that fibers are safe and effective in biological systems. We evaluated the antiviral activity of fibers loaded with either AZT or MVC using an *in vitro* TZM-bL assay. This model has previously been used to evaluate drug candidates for topical microbicides [11], [35]. Both the drug eluates from *in vitro* fiber release studies and drug-loaded fibers themselves were found to potently inhibit HIV compared to blank fibers. The IC50 values of unformulated ARV versus eluted ARV were of a same order of magnitude (**Fig.** **4a**). Our results suggest that fibers are able to release sufficient levels of drug in cDMEM to prevent HIV infection in TZM-bL cells over 48 hours, which is consistent with the release profiles obtained from *in vitro* release studies in VFS (**Fig. 4b**). Furthermore, these studies demonstrate that both drugs are in a bioavailable form after the electrospinning formulation process. The toxicity of fibers was measured with a macaque cervical explant model using histological examination and an MTT viability assay (**Fig.** **4**). A similar model has previously been used to evaluate the safety of microbicide candidates using human cervical explant tissue [36]–[38]. Tissue exposed to either 30∶70 or 70∶30 PLLA/PEO fibers was found to have similar epithelial layer integrity and cell viability compared with untreated control tissue, indicating the biological suitability of the polymer blends for additional studies in vaginal drug delivery.

Long-term shelf stability is another quality imperative to the design of effective dosage forms for multipurpose prevention. Ham *et* *al*. performed an extensive characterization of the long-term stability of pyrimidinedione vaginal film, measuring drug release, toxicity, and activity at specified time points over 12 months at both standard and elevated temperature and humidity [39]. We detected >95% of the initially-loaded AZT and MVC in fiber meshes stored at room temperature for at least five months and observed that fiber meshes retained similar appearance and texture over this period. While this data suggest that ARVs remain stable in PLLA/PEO fiber meshes under standard storage conditions, more comprehensive studies of drug stability are needed for the polymer-drug combinations chosen for future studies. We expect that the solid dosage form of drug-loaded fibers will be advantageous for long-term stability compared with semi-solid dosage forms like vaginal gels.

There is a great need to develop multipurpose prevention strategies that provide contraception in addition to protecting against STIs including HIV. Topically applied non-hormonal chemical contraceptives for pericoital use would address an important gap in contraception needs for women. While N-9 is a highly effective spermicide, the same detergent properties responsible for immobilizing sperm are also known to promote vaginal inflammation and increase the risk of STI infection [5], [40]. We first evaluated non-hormonal contraceptives described previously in the literature to be potent inhibitors of sperm function. We tested the function of FeGluc, Asc, and their mixture because these agents had been reported previously to potently inhibit sperm motility in solution and after elution from a vaginal ring [23], [41]. At the highest concentration tested, FeGluc alone inhibited motility of ∼37% of sperm within 2 minutes but did not result in complete spermiostasis (Table S1). Combining FeGluc with L-ascorbic acid (FeAsc) resulted in rapid spermiostasis in <30 s. However, we observed equally rapid spermiostasis upon treatment with L-ascorbic acid alone. We conclude that the reported spermicidal effect of FeGluc when combined with L-ascorbic acid was likely due to the decrease in pH upon dissolution of L-ascorbic acid (pH∼2), since pure L-ascorbic acid was just as effective as its mixture with iron. Indeed, L-ascorbic acid at a pH of 5.5 had no effect on sperm motility (Table S1). MBCD was also evaluated as a non-hormonal chemical contraceptive but showed no effect on sperm function at the concentrations tested. In addition, due to the high amounts of free cholesterol present in whole semen, we estimate that the high concentration of MBCD required to induce sperm capacitation would exceed the concentration that causes inflammation [24], [42].

We were motivated to evaluate GML's activity on sperm function based on its amphiphilic properties and its reported interaction with lipid bilayers [25]. GML, a glycerol ester of lauric acid that is used commonly as an emulsifier in foods and cosmetics, is regarded by the FDA as safe for topical use at doses up to 100 mg mL^−1^ [43]. Additionally, GML is inexpensive, possesses documented anti-inflammatory properties, and is antimicrobial for a number of vaginal pathogens [43], [44]. We show for the first time that GML potently reduces sperm motility in a dose-dependent manner (EC_50_∼0.005% wt/vol at 2 min) and significantly lowers sperm viability at concentrations equivalent to those used in recent microbicide studies with macaques (5% wt/vol) (**Fig.** **5**). Although its spermicidal mechanisms are as yet unknown, interference with signal transduction by incorporation into plasma membranes has been suggested as a mechanism for its antibacterial and anti-inflammatory properties [43], [45], [46] and may also be involved in sperm inhibition. Future studies should perform an in-depth characterization of how GML causes spermiostasis. Our findings add to the list of properties that make GML an attractive candidate for use in topical microbicides for multipurpose prevention, including anti-inflammatory mediated prevention of SIV infection in macaques [26] and the capacity to prevent bacterial infections [47]. The low aqueous solubility of GML (50–100 µg mL^−1^ at pH 7) precluded our ability to evaluate the activity of higher concentrations of GML on human sperm in whole semen, but provided a strong rationale to formulate GML in polymer fibers [43]. A dosage form that enhances the bioavailability of GML could potentially enhance the spermicidal potency of the compound.

The potential of GML to act as a surfactant has raised controversy within the microbicide field over its safety as a topical product. Schlievert et al. provided extensive characterization of the safety of 5% GML in KY warming gel for daily use in macaques for up to 12 weeks (n = 9) [43]. Using an MTT viability assay and histological examination of polarized cervical explants from macaques, we found that 30∶70 PLLA/PEO fibers loaded with 10% (wt/wt) GML were nontoxic *ex vivo* and had similar epithelial layer integrity to untreated controls (**Fig.** **4**). Nonoxynol-9 has been found to increase the frequency of genital lesions, which, it is thought, subsequently increase the risk of HIV infection [5]. In contrast, GML actually stabilizes eukaryotic cell membranes and reduces production of IL-8, an inflammatory cytokine. Furthermore, GML does not negatively impact the growth of lactobacilli or the production of lactic acid *in vivo* [43]. In contrast, nonoxynol-9 irritates and removes vaginal and cervical epithelial cells (**Fig.** **4c**) and disrupts the normal vaginal flora [48], [49]. While Moench et al. demonstrated that GML increased susceptibility to HSV-2 infection in mice, they acknowledged that the vaginal epithelium of medroxyprogesterone acetate treated mice is quite different from that of humans and nonhuman primates [50]. Depoprovera-treated murine vaginal lining is similar to endocervical columnar epithelium, which is much thinner and less robust than the non-keratinized stratified squamous epithelium of the ectocervix or vaginal wall in humans and non-human primates. Since HSV-2 infection may occur through the vulva, the vagina, or the cervix, the mouse model developed by Moench et al. may be too sensitive to draw conclusions about how GML may affect HSV-2 acquisition in humans or nonhuman primates. Despite the surfactant nature of GML, the compound has been shown to have several protective qualities, including the ability to neutralize the toxic effects of pathogenic gram-positive bacteria [25], [51]. Recently, Li et al. provided preliminary evidence that GML is not only safe for chronic use in rhesus macaques, but actually prevents SIV mucosal transmission with repeated high dose challenge [26].

In addition to the chemical inhibition provided by GML, we also demonstrated that blank fibers block sperm migration in a transwell assay (**Fig.** **5** **, Table S2**). Since the electrospun fibers used as a physical barrier were five times as thick as the tissue culture inserts, the tissue culture inserts cannot serve as a control to discern why the fiber meshes formed a functional barrier against sperm transport. Nevertheless, they do still provide a control to demonstrate that the sperm were motile and capable of traversing a membrane with 3 μm diameter pores. Relying upon a porous mesh to block sperm penetration differs from current barrier approaches, which rely on nonporous materials to block sperm entry into the cervix [52]–[54]. Our results showed that a porous, tortuous mesh fabricated by electrospinning could efficiently block sperm entry. This suggests that, if fabricated in the appropriate geometry and given the appropriate mechanical strength, electrospun fibers may serve as an effective barrier contraceptive. While the materials we presented are not yet suitable to be turned into a final barrier device, analysis of their mechanical properties does shed some light on potential product configurations. In particular, the Young's moduli of electrospun materials made from 70∶30 PLLA/PEO fibers with 1% (wt/wt) MVC were around 50–100 MPa, and electrospun meshes withstood at least 50% extensional strain before failure (**Fig. S12**). For comparison, latex rubber condoms have a Young's modulus of approximately 2 MPa and can withstand inflation to volumes greater than 20 L [55]. Dapivirine films have a tensile modulus of 5.4 to 7.8 MPa [13]. Based on the mechanical properties of these electrospun materials, it is unlikely that they would be effective as a condom-like device, but they may be suitable as devices similar to vaginal sponges or diaphragms.

We have presented a versatile platform for topical drug delivery to the lower female reproductive tract. The electrospun fiber meshes were fabricated in geometries suitable for intravaginal drug delivery, and we showed that our fibers incorporate agents with differing aqueous solubility and mechanisms of action against HIV-1, HSV-2, or sperm. Layering or co-electrospinning drug-loaded fibers may create composite materials that are multifunctional by virtue of simultaneous delivery of multiple agents with different mechanisms of action. In particular, fibers incorporating inhibitors of viral reverse transcriptase and CCR5 binding prevented HIV infection *in vitro*. We also screened non-hormonal chemical contraceptive alternatives to N-9 and identified a novel function of GML to inhibit sperm motility and reduce sperm viability. In addition to chemically inhibiting sperm function, fibers also physically inhibit sperm penetration by creating a tortuous path that sperm cannot navigate. This system is expected to provide enhanced coverage of the mucosal tissue and vaginal rugae by covering both the vaginal wall and the cervix, supported by the extent of coverage demonstrated when fiber meshes were applied to mice. We envision that the fiber meshes could be inserted simply with a tampon applicator, rendering it discreet, female-controlled, and wholly reversible. Further research will be conducted to investigate the mechanical properties of the fibers and explore other methods to modulate drug release. The functional combination offered by our drug-eluting fibers cannot be accomplished with any single technology currently in the development pipeline. To our knowledge, this research represents the first application of electrospinning to vaginal drug delivery. We envision other applications for drug-eluting fibers along with prevention technology, including mucosal vaccine delivery, STI treatment, rectal microbicides, and other reproductive health applications.

## Materials and Methods

### Ethics statement

All animals were obtained and cared for in accordance with the University of Washington Institutional Animal Care and Use Committee (IACUC) guidelines, and animal studies were approved by the University of Washington IACUC. Human semen samples were obtained according to guidelines approved by the Institutional Review Board of the Human Subjects Division at the University of Washington. All subjects provided written, informed consent prior to participation, and the study protocol was approved by the University of Washington Institutional Review Board.

### Polymer preparation

Polymers used for electrospinning included poly(L-lactide) with an inherent viscosity of 0.90–1.20 dL g^−1^ (MW∼117 kDa) (Lactel Absorbable Polymers), poly(ethylene oxide) with MW 100 kDa (Sigma-Aldrich), polycaprolactone of M_n_ 70–90 kDa (Sigma-Aldrich), and acid terminated poly(D, L-lactide) of M_w_ 18–24 kDa (Sigma Aldrich). Maraviroc was obtained from the NIH AIDS Research & Reference Reagent Program, Division of AIDS, NIAID, NIH. 3′-Azido-3′-deoxythymidine, methyl-β-cyclodextrin (M_n_ = 1310), acyclovir, iron(II) D-gluconate, and L-ascorbic acid were purchased from Sigma-Aldrich. Glycerol monolaurate was purchased from MP Biomedicals, LLC. VFS was made according to methods described by Owen and Katz, *et* *al* [56]. Potassium hydroxide, calcium hydroxide, lactic acid, acetic acid, and glycerol were purchased from Fisher Scientific. Bovine serum albumin, urea, and glucose were obtained from Sigma-Aldrich. Sodium chloride was purchased from Mallinckrodt Chemicals. The pH for VFS was adjusted to 4.2 with HCl and filter sterilized.

### Electrospinning

PLLA and PEO were dissolved at 5%, 10%, 15% (wt/vol) in mixtures of 1∶1 or 3∶1 (vol/vol) chloroform (EMD Chemicals) and 2,2,2-trifluoroethanol (Sigma-Aldrich). PCL was dissolved at 10% and 15% (wt/vol) in 2,2,2-trifluoroethanol. PDLLA and PLLA were dissolved at 15% (wt/vol) in 1∶1 chloroform and 2,2,2-trifluoroethanol. Drugs were mixed with polymers at 1 or 10% (wt/wt) prior to addition to solvent. Polymer solutions were loaded into glass gastight syringes (National Scientific) and set into a precision syringe pump (KD Scientific Inc.). Unless otherwise specified, fibers were produced with the following parameters. We dispensed 500 µL at a flow rate of 5 µL min^−1^ through a gauge 22 stainless steel dispensing needle (Integrated Dispensing Solutions, Inc.) that was clamped to +15 kV using a high voltage generator (Gamma High Voltage Research). The aluminum mandrel collector was machined at the University of Washington to have a diameter of 1.27 cm. The collector was placed 12 cm horizontally from the tip of the needle and set to 3,000 r.p.m. (linear rotational speed of 200 cm s^−1^ at the surface of the collector) with a 5.08 cm horizontal travel at a speed of 2.54 cm s^−1^. A copper or graphite brush electrically grounded the mandrel. 3.4 μm 70∶30 PLLA/PEO fibers were produced as above except polymers were dissolved at 25% (wt/vol) and spun at 100 μL min^−1^ at 1,200 rpm. 1.5 μm 70∶30 PLLA/PEO fibers were produced as 3.4 μm fibers except a 20% (wt/vol) solution of polymer was used. PDLLA/PLLA fibers were produced as above except for the flow rate and mandrel speed, which were 100 μL min^−1^ and 1,200 rpm, respectively. PCL fibers were produced by dispensing 500 μL at a flow rate of 100 μL min^−1^ from a 25 G needle clamped to +12 kV voltage and set 8 cm from the collector, which was rotating at 1,200 rpm. Electrospun meshes were removed from the collector and lyophilized for at least 24 h before imaging or use in biological assays.

### Material characterization

Polymer recovery was determined by dividing the mass of polymer removed from the mandrel by the theoretical mass of polymer and drug present in 500 µL of polymer solution. Meshes were sputtered with gold/palladium for 70 s and imaged with SEM (Sirion or JSM-7000F, JEOL Ltd.) at 500× and 5,000× magnification. Two scanning electron microscopes were used to complete imaging: a Sirion SEM at the University of Washington Nanotechnology Facility (funded by National Science Foundation), and a JSM-7000F SEM (JEOL Ltd.) at the Materials Science and Engineering Department at the University of Washington. Fiber size was determined in ImageJ (NIH) by measuring fibers that intersected a diagonal line drawn across each 5,000x micrograph to eliminate bias. At least 100 fibers from at least three separate micrographs were measured for each sample. Degradation in VFS was analyzed by placing 5 mg samples of mesh in 6 mL of VFS at 37°C for up to 15 days. Samples were removed at times up to 15 days, immersed gently in distilled water, inverted three times to remove salts, and lyophilized. The fibers were massed again, and percent mass loss was calculated as (original mass – current mass)/(original mass) ×100%. Fiber mesh thickness was measured using calipers. Dog bone shaped samples were cut from collected meshes with a D1708-96-MET die (ODC Tooling and Molds) such that the long axis of the dog bone corresponded to the circumferential direction of the mandrel collector. Uniaxial tensile testing was performed with an Instron model 5543 instrument and model 2712-03 grippers (Instron). Samples were stretched at a rate of 10 mm/min until failure. Young's modulus was estimated by fitting stress-strain curves with a line for 0–15% of maximum stress.

### Drug release and loading

Triplicate samples of mesh approximately 10 mg each containing AZT or MVC were placed into 6 mL glass vials, immersed in 6 mL of VFS, and incubated at 37°C on an orbital shaker at 200 r.p.m. At set time points (1 h, 4 h, 8 h, 12 h, 24 h, 48 h), 500 µL of buffer was removed and replaced with fresh VFS. A Shimadzu Prominence LC20AD UV-HPLC system equipped with a Phenomenex Luna C18 column (5 µm, 250×4.6 mm) and LCSolutions software were used to quantify drug levels in samples. Methods for MVC were based on those as described [57]. The mobile phase consisted of HPLC grade 0.01 M KH_2_PO_4_ buffer and acetonitrile (60∶40, vol/vol) (EMD Chemicals) at isocratic flow rate of 1.0 mL min^−1^ for 10 min. Column oven temperature was 25°C. Standards were made in VFS, with linearity established from 0.001 to 0.02 mg mL^−1^ with 20 µL injection volume. MVC was detected at 193 nm with a retention time of 3.1 to 4.1 min. AZT was detected using an isocratic mobile phase was composed of HPLC grade water with 0.045% trifluoroacetic acid and acetonitrile with 0.036% trifluoroacetic acid (72∶28) at a flow rate of 1.0 mL min^−1^ for 15 min, with column oven temperature of 30°C. AZT was detected at 265 nm at retention time of 4.4 min. Standards were prepared in water, with a linear range from 0.001 to 0.5 mg mL^−1^ with 10 µL injection volume. GML release from fiber meshes was detected using thin-layer chromatography (TLC). 10 mg pieces of either 30∶70 PLLA/PEO or 70∶30 PLLA/PEO with 10% (wt/wt) GML were added in triplicate to 6 mL of PBS at pH 4.2. Samples were incubated at 37**°**C, nd 500 μL ere removed at periodic intervals and replaced with fresh PBS (pH 4.2) or 48 h. μL of release media (n = 3) were added onto duplicate TLC plates. After drying, plates were baked at 100**°**C for 10 min, then allowed to cool to room temperature. Plates were then immersed in 0.025% (wt/vol) Coomassie blue (Fisher) in 20% (vol/vol) methanol for 10 s and allowed to dry for 1 h. Plates were then digitized using a scanner. Drug loading and stability was measured using fibers stored at room temperature (19–22°C) for at least five months. 10 mg pieces of fiber mesh were dissolved in 2.5 mL acetonitrile, centrifuged for 10 min at 10,000 g, and added to 0.01 M KH_2_PO_4_ buffer or water at a 1∶1 ratio for MVC or AZT fibers, respectively. UV-HPLC was used to quantify amount of drug in samples as previously described. Encapsulation efficiency was calculated as the amount of drug in drug-loaded fibers relative to the amount of drug detected in dissolved blank fibers spiked at 1% (wt/wt) drug loading.

### Mouse fiber coverage study

Two eight-week old female Balb/cByJ mice (Jackson Laboratories) were cycled with injections of medroxyprogesertone acetate (Greenstone LLC) four days prior to fiber insertion. Fiber meshes of dimensions 2×2 cm were folded around an applicator and inserted into the mouse vagina. The control mouse received blank 30∶70 PLLA/PEO fibers, and the experimental mouse received 30∶70 PLLA/PEO fibers electrospun with 1% (w/w) indocyanine green (Sigma-Aldrich). Mice were anesthetized during the procedure with isoflurane administered through nose cones. Mice were sacrificed after 30 minutes, and reproductive tracts were excised for imaging. Fiber meshes were removed after dissection and imaged with excised reproductive tracts. A Xenogen in vivo imaging system (IVIS) was used to measure fluorescence at 745/820 nm as a surrogate for fiber coverage.

### HIV inhibition assay

TZM-bl cells and HIV-1 BaL isolate were obtained from the NIH AIDS Research and Reference Reagent Program, Division of AIDS, NIAID, NIH (http://www.aidsreagent.org/). TZM-bL cells, a derived HeLa cell line that expresses CD4, CCR5, and CXCR4 [58]–[61], were added to black 96-well plates (Corning, Corning, NY) with Dulbecco's Modified Eagle Medium (DMEM) (Gibco Life Technologies) with 10% fetal bovine serum (Hyclone), 1% 100X penicillin/streptomycin (Invitrogen), and 1% 200 mM L-glutamine (Invitrogen) with 50 µL/well at a density of 5,000 cells/well. Cells were incubated in 5% CO_2_ and 37°C for 24 h prior to exposure to drugs or fibers. Fibers were sterilized by UV irradiation for 2 h (1 h per side). Treatments were added in 50 µL volumes. For the HIV-infectious inhibition assay, 100 µL of HIV-BaL (240 TCID/well) was added to wells 1 h after drug treatment. Media was removed from cells after 48 h post-treatment, and 100 µL of phosphate buffered saline (Gibco Life Technologies) and 100 µL of Bright-Glo Luciferase reagent (Promega) were added to wells. Infectious activity was quantified by measuring luminescence on a plate reader (Tecan). IC_50_ values of drug compounds were estimated using sigmoidal regression and bootstrapping in MATLAB version 7.11 (Mathworks).

### Explant toxicity assay

Macaque ectocervical tissues (Tissue Banking and Distribution Program, University of Washington National Primate Research Center) were processed for polarized explant cultures in duplicate on the day of surgery as previously described [36]–[38]. Briefly, a circular tissue punch was inserted through the transwell membrane with the luminal side up. The edges around the explant were sealed with Matrigel^TM^ (BD Biosciences, San Jose, CA). A 0.6 mm diameter disc of either 30∶70 PLLA/PEO, 70∶30 PLLA/PEO, or 30∶70 PLLA/PEO with 10% (wt/wt) GML fiber was placed on the apical side of the tissue with 200 µL of culture media (DMEM with 10% fetal bovine serum, 1% 100X penicillin/streptomycin, and 1% 200 mM L-glutamine). For controls, explants were untreated (culture media) or treated with a 0.4% dilution of nonoxynol-9 (N-9) gel. The explant cultures were maintained at 37°C in a 5% CO_2_ atmosphere. After 18–24 h, the explants were washed and one of each duplicate was incubated in RPMI containing 250 µg/ml MTT [1-(4,5-dimethylthiazol-2-yl)-3,5-diphenylformazan] for 4 h. The explants were removed and placed in 1 mL of methanol overnight to extract the formazan dye produced by live tissue. The next day, the explants were removed from methanol and placed on a marked paper towel to dry and be weighed. The color extracted in the methanol was read for optical density at 595 nm. The percent viability of the treated explants was determined by correcting the optical density (OD) with the weight of the corresponding explant. The other explant was frozen in an embedding medium (Tissue-Tek, Sakura Finetek USA Inc., CA) and processed for histology by cryosectioning and hematoxylin-eosin staining.

### Sperm motility and viability assays

Sperm was obtained from two donors for sperm motility experiments. A third donor was recruited for sperm viability assays with glycerol monolaurate. Swim out sperm were obtained as described previously [62]. Briefly, we placed 0.5 mL aliquots of semen below 3 mL of Ham's F-10 media (Sigma-Aldrich) with 0.5% human serum albumin (Sigma-Aldrich) for 75 min in 5% CO_2_ and 37°C. The aspirate, enriched for motile sperm, was centrifuged at 300 RCF for five min and resuspended in fresh Ham's F-10 to a concentration of 20×10^6^ sperm mL^−1^. The effect of drug dilutions on sperm motility were performed both in whole semen and in swimout sperm by adding 5 µL each of sperm and drugs to a slide and observing sperm motility with phase contrast either at 200× and 37°C (ECLIPSE Ti, Nikon), or by adding 200 µL of drug to 100 µL of semen and quantifying sperm motility with computer aided motility analysis for up to 7 min after the addition of drug. Multiple media only controls were run to ensure that any observed change in motility was minimally dependent upon time since ejaculation. Sperm viability was measured by adding 20 µL of semen to 20 µL of Trypan blue (Sigma-Aldrich) and counting 100 live or dead sperm based on head staining after a 10 min incubation using 1000× brightfield microscopy.

### Sperm migration assay

Millicell cell culture insert membranes (Millipore) with 3 µm pores were removed with forceps and replaced with square pieces of electrospun mesh. The mesh was attached to the inserts by applying firm pressure with a gloved finger. The thicknesses of cell culture insert membranes and electrospun meshes were measured using a micrometer. Modified and unmodified inserts were placed in a 12 well plate. Swimout sperm were diluted 1∶10 in Ham's F-10 with no protein. 600 µL of Ham's F-10 was added to each of the twelve wells, and 400 µL of diluted sperm was added to each insert. Sperm were incubated for 2 h at 37°C and 5% CO_2_. The solutions from the inner and outer chambers of the wells were aspirated and used for counting to measure sperm concentration in media inside and outside of the inserts. Sperm were fixed by dipping membranes into ice cold ethanol and were then lyophilized for 24 h. Meshes were imaged using SEM with the same settings used to image blank meshes.

### Statistical methods

Fiber diameters were plotted as geometric mean with 95% CI and reported in writing as geometric mean with standard deviation. As fiber diameters had lognormal distributions, they were transformed by taking the log of the diameters in nm prior to hypothesis testing. One-way ANOVA and Bonferroni t tests were used to compare the diameters of log-transformed fiber diameters. Drug release was reported as mean with standard deviation, and 6 d release values were compared by one-way ANOVA with Bonferroni t tests or compared to zero release with a one sample t test. Differences in amount of drug release were reported as mean difference with 95% confidence interval. Sperm viability in the presence of GML was analyzed using one-way ANOVA and a Bonferroni t test. The numbers of sperm counted in the transwell migration assay were reported as mean and standard deviation of log transformed data, except in the case where the number of sperm detected was zero. We used two-sided tests at a significance level of α = 0.05 for all hypothesis testing. Actual p values are reported unless p<0.0001.

## Supporting Information

Figure S1 Videos show sperm motility at 5 min after addition of GML, PBS, or Nonoxynol 9.
**Fiber diameters depend upon polymer viscosity, composition, and solvent.** Geometric mean fiber diameters with 95% confidence intervals are shown for 70∶30 PLLA/PEO fibers (red) and 30∶70 PLLA/PEO fibers (blue) made from polymers dissolved in either 1∶1 or 3∶1 (vol/vol) chloroform/2,2,2-trifluoroethanol). Geometric mean fiber diameter was found to vary significantly between all groups based on the concentration of polymer in the electrospinning solution (solid  = 15% wt/vol, dashed  = 10% wt/vol, dots  = 5% wt/vol) (P<0.0001), except as indicated.
(TIFF)

Figure S2 **Material efficiency was positively correlated with % wt/vol of polymer in solution for most polymer blends.** Here we define material efficiency as the percent polymer recovered from the mandrel by mass. For 30∶70 PLLA/PEO blends, material efficiency increased with the % wt/vol of polymer in solution. Material efficiency is important for cost effectiveness. Based on these results, we chose the 15% (wt/vol) 70∶30 PLLA/PEO in 1∶1 chloroform/TFE and 15% wt/vol 30∶70 PLLA/PEO in 3∶1 chloroform/TFE as the base formulations for all further work with drug encapsulation.
(TIF)

Figure S3 **Electrospun fibers can be made sufficiently thick to be easily pushed out of a tampon applicator.** The mesh device can be loaded into standard packaging (top and bottom images on the left), and resembles a tampon once opened (2^nd^ to left, top – mesh, bottom – tampon). The device is delivered by pushing the inner cardboard tube through the outer cardboard tube of the applicator, revealing the fiber mesh, which would then hydrate and release active agents (3^rd^ to left, top – mesh, bottom – tampon). A side-by-side comparison of a tampon and the tubular mesh reveals comparable geometry (right).
(TIF)

Figure S4 **Electrospinning does not alter drug retention time with HPLC.** (**a**) Representative chromatogram for AZT. (**b**) Representative chromatogram for MVC. Peak shapes and retention times do not change for unformulated drugs (gray lines) compared with drugs released from fibers (black lines).
(TIF)

Figure S5 **Drug incorporation into 30∶70 and 70∶30 PLLA/PEO electrospun fibers can alter fiber diameter distributions.** Geometric mean fiber diameters with 95% confidence intervals are shown for 70∶30 PLLA/PEO fibers (red) and 30∶70 PLLA/PEO fibers (blue) incorporating MVC, AZT, ACV, GML, MBCD, or Fe/Asc compared to blank fibers.
(TIFF)

Figure S6 **Agent incorporation into fibers influences material efficiency.** Incorporating drugs into 30∶70 and 70∶30 PLLA/PEO blends (blue and red, respectively) affected polymer recovery. Interestingly, MVC dramatically increased material efficiency for 70∶30 PLLA/PEO fibers.
(TIFF)

Figure S7 **Fiber morphology and alignment for antiviral compounds ACV, MVC, and AZT.** Increased alignment of 70∶30 PLLA/PEO fibers is apparent in 1% MVC samples. Increased alignment of 30∶70 PLLA/PEO fibers is apparent in 1% AZT samples. Differences in fiber diameter, previously shown in Fig. S5, are also apparent.
(TIF)

Figure S8 **Fiber morphology and alignment for contraceptive compounds GML, Fe/Asc, and MBCD.** Increased alignment of 70∶30 PLLA/PEO fibers is apparent in 10% GML samples. Increased alignment of 30∶70 PLLA/PEO fibers is apparent in 10% MBCD samples. Differences in fiber diameter, previously shown in Supporting Fig. S5, are also apparent.
(TIF)

Figure S9 **Fiber diameters of 70∶30 PLLA/PEO with 1% (w/w) MVC.** Geometric mean fiber diameters with 95% confidence intervals are displayed for 70∶30 PLLA/PEO fibers with 1% (w/w) MVC made by varying polymer concentration and electrospinning parameters. Geometric mean fiber diameters of all three mesh types are significantly different from each other (p<0.0001). These fibers correspond to cumulative release curves displayed in Fig. 3b.
(TIFF)

Figure S10 **Morphology of additional polymeric fibers incorporating 1% (wt/wt) MVC.** Scanning electron micrographs of fibers made from different polymers incorporating 1% (wt/wt) MVC are shown, including 70∶30 PLLA/PEO of three fiber diameters (560 nm, 1.5 μm, and 3.4 μm), 99∶1 PLLA/PEO, PLLA, 25∶75 PDLLA/PLLA, 50∶50 PDLLA/PLLA, and PCL of two fiber diameters (371 nm and 1.3 μm). These fibers correspond to cumulative release curves displayed in Fig. 3.
(TIF)

Figure S11 **Fibers are not cytotoxic to TZM-bl cells.** Cytotoxicity was tested using the CellTiter-BlueTM cell viability assay by culturing TZM-bL cells with disks of blank and drug-loaded meshes (n = 3) for 48 h. Fluorescence signals for each condition are plotted as log-transformed values. Media-only treated cells and fiber-treated cells show significantly less cytotoxicity than bleach-treated cells (p<0.05).
(TIFF)

Figure S12 **Flow rate influences modulus of PCL fibers incorporating 1% (w/w) MVC.** Moduli of PCL fiber meshes made from electrospinning at varying flow rates (5, 50, and 100 μL/min) were measured. The modulus of PCL fibers electospun at a flow rate of 50 μl/min is significantly different than the modulus of PCL fibers electrospun at 5 μL/min (p<0.05).
(TIFF)

Video S1 **Sperm in PBS.** 10 uL of PBS were added to 10 uL of swimout sperm and then a glass coverslip was added to the sample while imaging at 100x magnification. The edges of the coverslip were sealed with clear nail polish to prevent streaming. Shown in this video are the PBS-treated sperm after 5 minutes of imaging. The sperm are clearly still motile.
(AVI)

Video S2 **Sperm in nonoxynol-9.** 10 uL of 0.4% wt/vol nonoxynol-9 were added to 10 uL of swimout sperm and then a glass coverslip was added to the sample while imaging at 100x magnification. The edges of the coverslip were sealed with clear nail polish to prevent streaming. Shown in this video are the nonoxynol-9-treated sperm after 5 minutes of imaging. The sperm are clearly completely immotile. Loss of motility was instant following addition of nonoxynol-9.
(AVI)

Video S3 **Sperm in 0.5% wt/vol GML.** 10 uL of 0.5% wt/vol GML were added to 10 uL of swimout sperm and then a glass coverslip was added to the sample while imaging at 100x magnification. The edges of the coverslip were sealed with clear nail polish to prevent streaming. Shown in this video are the GML-treated sperm after 5 minutes of imaging. The sperm are clearly immotile. Loss of motility was complete in under 4 minutes.
(AVI)

Video S4 **Sperm in 0.05% wt/vol GML.** 10 uL of 0.05% wt/vol GML were added to 10 uL of swimout sperm and then a glass coverslip was added to the sample while imaging at 100x magnification. The edges of the coverslip were sealed with clear nail polish to prevent streaming. Shown in this video are the GML-treated sperm after 5 minutes of imaging. The sperm are clearly immotile. Loss of motility was complete in under 5 minutes.
(AVI)

Table S1 **GML inhibits sperm motility in swimout sperm at 0.05 and 0.5% (wt/vol).** We tested the contraceptive agents for their ability to inhibit sperm motility in a dose response motility experiment. Equal amounts of drug solution and human swimout sperm were combined on a glass slide. Changes in sperm motility over 2–5 min were recorded using a microscope and video recording system. Time point controls with PBS were performed to ensure drug effects on motility were independent of sperm incubation time. PBS percent motility at 2 min reflects average over all PBS control measurements, with range from 72%–100%.
(DOC)

Table S2 **Electrospun fibers effectively block sperm migration in a transwell assay.** We tested the ability of electrospun fiber mats to block sperm migration in a transwell assay. 30∶70 PLLA/PEO fibers without drug were used to replace existing membranes in transwell cups. Motile sperm collected by swimout into media were added to the upper chamber (inside of the cup) and allowed to attempt to enter the bottom chamber for 2 hours. Sperm were then counted in both chambers to assess if sperm could penetrate the meshes.
(DOC)

## References

[1] Glasier A, Gülmezoglu AM, Schmid GP, Moreno CG, Van Look PF (2006) Sexual and reproductive health: a matter of life and death. The Lancet 368: 1595–1607 doi:10.1016/S0140-6736(06)69478-6 10.1016/S0140-6736(06)69478-6 17084760

[2] CAMI Multipurpose Prevention Technologies for Reproductive Health: 2011 Symposium (2012) 1–24.

[3] Blish C, Baeten J (2011) Hormonal Contraception and HIV-1 Transmission. American Journal of Reproductive Immunology 65: 302–307. 21087338 10.1111/j.1600-0897.2010.00930.x PMC3058314

[4] Baeten JM, Lavreys L, Overbaugh J (2007) The Influence of Hormonal Contraceptive Use on HIV-1 Transmission and Disease Progression. Clinical Infectious Diseases 45: 360–369 doi:10.1086/519432 17599316 10.1086/519432

[5] Van Damme L, Ramjee G, Alary M, Vuylsteke B, Chandeying V, et al (2002) Effectiveness of COL-1492, a nonoxynol-9 vaginal gel, on HIV-1 transmission in female sex workers: a randomised controlled trial. The Lancet 360: 971–977 doi:10.1016/S0140-6736(02)11079-8 10.1016/s0140-6736(02)11079-8 12383665

[6] Friend DR, Doncel GF (2010) Combining prevention of HIV-1, other sexually transmitted infections and unintended pregnancies: Development of dual-protection technologies. Antiviral Research 88 Supplement: S47–S54–doi:10.1016/j.antiviral.2010.09.005 21109068 10.1016/j.antiviral.2010.09.005

[7] Minnis A, Padain N (2005) Effectiveness of female controlled barrier methods in preventing sexually transmitted infections and HIV: current evidence and future research directions. Sexually transmitted infections 81: 193–200. 15923284 10.1136/sti.2003.007153 PMC1744969

[8] Malcolm RK, Edwards K-L, Kiser P, Romano J, Smith TJ (2010) Advances in microbicide vaginal rings. Antiviral Research 88 Supplement: S30–S39 doi:10.1016/j.antiviral.2010.09.003 21109066 10.1016/j.antiviral.2010.09.003

[9] Garg S, Goldman D, Krumme M, Rohan LC, Smoot S, et al (2010) Advances in development, scale-up and manufacturing of microbicide gels, films, and tablets. Antiviral Research 88: S19–S29 doi:10.1016/j.antiviral.2010.09.010 21109064 10.1016/j.antiviral.2010.09.010

[10] Karim Q, Karim SSA, Frohlich JA, Grobler AC, Baxter C, et al (2010) Effectiveness and Safety of Tenofovir Gel, an Antiretroviral Microbicide, for the Prevention of HIV Infection in Women. Science 329: 1168. 20643915 10.1126/science.1193748 PMC3001187

[11] Herrera C, Cranage M, McGowan I, Anton P, Shattock RJ (2009) Reverse Transcriptase Inhibitors as Potential Colorectal Microbicides. Antimicrobial Agents and Chemotherapy 53: 1797–1807 doi:10.1128/AAC.01096-08 19258271 10.1128/AAC.01096-08 PMC2681527

[12] Singer R, Derby N, Rodriguez A, Kizima L, Kenney J, et al (2011) The Nonnucleoside Reverse Transcriptase Inhibitor MIV-150 in Carrageenan Gel Prevents Rectal Transmission of Simian/Human Immunodeficiency Virus Infection in Macaques. Journal of Virology 85: 5504–5512 doi:10.1128/JVI.02422-10 21411526 10.1128/JVI.02422-10 PMC3094984

[13] Akil A, Parniak M, Dezzutti C, Moncla B, Cost M, et al (2011) Development and characterization of a vaginal film containing dapivirine, a non-nucleoside reverse transcriptase inhibitor (NNRTI), for prevention of HIV-1 sexual transmission. Drug Delivery and Translational Research 1: 209–222 doi:10.1007/s13346-011-0022-6 22708075 10.1007/s13346-011-0022-6 PMC3375737

[14] Malcolm K, Woolfson D, Russell J, Andrews C (2003) In vitro release of nonoxynol-9 from silicone matrix intravaginal rings. Journal of Controlled Release 91: 355–364 doi:10.1016/S0168-3659(03)00260-8 12932713 10.1016/s0168-3659(03)00260-8

[15] Malcolm RK (2005) Long-term, controlled release of the HIV microbicide TMC120 from silicone elastomer vaginal rings. Journal of Antimicrobial Chemotherapy 56: 954–956 doi:10.1093/jac/dki326 16155060 10.1093/jac/dki326

[16] Szentivanyi A, Chakradeo T, Zernetsch H, Glasmacher B (2011) Electrospun cellular microenvironments: Understanding controlled release and scaffold structure. Advanced Drug Delivery Reviews 63: 209–220 doi:10.1016/j.addr.2010.12.002 21145932 10.1016/j.addr.2010.12.002

[17] Teo W-E, Inai R, Ramakrishna S (2011) Technological advances in electrospinning of nanofibers. Science and Technology of Advanced Materials 12: 013002 doi:10.1088/1468-6996/12/1/013002 27877375 10.1088/1468-6996/12/1/11660944 PMC5090397

[18] Ratner B, Hoffman A, Schoen F, Lemons J (1996) Biomaterials science: an introduction to materials in medicine. San Diego: Academic Press. 297-308 p.

[19] Anderson JM, Shive MS (1997) Biodegradation and biocompatibility of PLA and PLGA microspheres. Advanced Drug Delivery Reviews 28: 5–24 doi:10.1016/S0169-409X(97)00048-3 10837562 10.1016/s0169-409x(97)00048-3

[20] Auras RA, Lim L-T, Selke SEM, Tsuji H (2010) Poly(Lactic Acid): Synthesis, Structures, Properties, Processing, and Applications. John Wiley & Sons. 356-357 p.

[21] Teo W-E, He W, Ramakrishna S (2006) Electrospun scaffold tailored for tissue-specific extracellular matrix. Biotechnology Journal 1: 918–929 doi:10.1002/biot.200600044 16941439 10.1002/biot.200600044

[22] Yohe ST, Colson YL, Grinstaff MW (2012) Superhydrophobic Materials for Tunable Drug Release: Using Displacement of Air To Control Delivery Rates. Journal of the American Chemical Society 134: 2016–2019 doi:10.1021/ja211148a 22279966 10.1021/ja211148a PMC3878812

[23] Saxena B, Singh M, Gospin R, Chu C, Ledger W (2004) Efficacy of nonhormonal vaginal contraceptives from a hydrogel delivery system. Contraception 70: 213–9. 15325890 10.1016/j.contraception.2004.02.015

[24] Zidovetzki R, Levitan I (2007) Use of cyclodextrins to manipulate plasma membrane cholesterol content: evidence, misconceptions and control strategies. Biochimica et biophysica acta 1768: 1311–24. 17493580 10.1016/j.bbamem.2007.03.026 PMC1948080

[25] Peterson ML, Schlievert PM (2006) Glycerol Monolaurate Inhibits the Effects of Gram-Positive Select Agents on Eukaryotic Cells. Biochemistry 45: 2387–2397 doi:10.1021/bi051992u 16475828 10.1021/bi051992u PMC2553893

[26] Li Q, Estes J, Schlievert P, Duan L, Brosnahan A, et al (2009) Glycerol monolaurate prevents mucosal SIV transmission. Nature 458: 1034. 19262509 10.1038/nature07831 PMC2785041

[27] Dorr P, Westby M, Dobbs S, Griffin P, Irvine B, et al (2005) Maraviroc (UK-427,857), a Potent, Orally Bioavailable, and Selective Small-Molecule Inhibitor of Chemokine Receptor CCR5 with Broad-Spectrum Anti-Human Immunodeficiency Virus Type 1 Activity. Antimicrob Agents Chemother 49: 4721–4732 doi:10.1128/AAC.49.11.4721-4732.2005 16251317 10.1128/AAC.49.11.4721-4732.2005 PMC1280117

[28] Wald A, Link K (2002) Risk of Human Immunodeficiency Virus Infection in Herpes Simplex Virus Type 2–Seropositive Persons: A Meta-analysis. The Journal of Infectious Diseases 185: 45–52 doi:10.1086/338231 11756980 10.1086/338231

[29] Graver MA, Wade JJ (2011) The role of acidification in the inhibition of Neisseria gonorrhoeae by vaginal lactobacilli during anaerobic growth. Annals of Clinical Microbiology and Antimicrobials 10: 8 doi:10.1186/1476-0711-10-8 21329492 10.1186/1476-0711-10-8 PMC3045876

[30] Yu D-G, Yang J-M, Branford-White C, Lu P, Zhang L, et al (2010) Third generation solid dispersions of ferulic acid in electrospun composite nanofibers. International Journal of Pharmaceutics 400: 158–164 doi:10.1016/j.ijpharm.2010.08.010 20713138 10.1016/j.ijpharm.2010.08.010

[31] Yu D-G, Branford-White C, White K, Li X-L, Zhu L-M (2010) Dissolution Improvement of Electrospun Nanofiber-Based Solid Dispersions for Acetaminophen. AAPS PharmSciTech 11: 809–817 doi:10.1208/s12249-010-9438-4 20446072 10.1208/s12249-010-9438-4 PMC2902305

[32] Kieweg SL, Katz DF (2006) Squeezing Flows of Vaginal Gel Formulations Relevant to Microbicide Drug Delivery. Journal of Biomechanical Engineering 128: 540 doi:10.1115/1.2206198 16813445 10.1115/1.2206198

[33] Cui W, Li X, Zhu X, Yu G, Zhou S, et al (2006) Investigation of Drug Release and Matrix Degradation of Electrospun Poly(dl-lactide) Fibers with Paracetanol Inoculation. Biomacromolecules 7: 1623–1629 doi:10.1021/bm060057z 16677047 10.1021/bm060057z

[34] Forbes CJ, Lowry D, Geer L, Veazey RS, Shattock RJ, et al (2011) Non-aqueous silicone elastomer gels as a vaginal microbicide delivery system for the HIV-1 entry inhibitor maraviroc. Journal of Controlled Release 156: 161–169 doi:10.1016/j.jconrel.2011.08.006 21864598 10.1016/j.jconrel.2011.08.006 PMC3220778

[35] Ham A, Cost M, Sassi A, Dezzutti C, Rohan L (2009) Targeted Delivery of PSC-RANTES for HIV-1 Prevention using Biodegradable Nanoparticles. Pharmaceutical Research 26: 502–511 doi:10.1007/s11095-008-9765-2 19002569 10.1007/s11095-008-9765-2 PMC4243299

[36] Abner SR, Guenthner PC, Guarner J, Hancock KA, Cummins JE, et al (2005) A Human Colorectal Explant Culture to Evaluate Topical Microbicides for the Prevention of HIV Infection. J Infect Dis 192: 1545–1556 doi:10.1086/462424 16206069 10.1086/462424

[37] Rohan LC, Moncla BJ, Kunjara Na Ayudhya RP, Cost M, Huang Y, et al (2010) In Vitro and Ex Vivo Testing of Tenofovir Shows It Is Effective As an HIV-1 Microbicide. PLoS ONE 5: e9310 doi:10.1371/journal.pone.0009310 20174579 10.1371/journal.pone.0009310 PMC2824823

[38] Cummins JE, Guarner J, Flowers L, Guenthner PC, Bartlett J, et al (2007) Preclinical Testing of Candidate Topical Microbicides for Anti-Human Immunodeficiency Virus Type 1 Activity and Tissue Toxicity in a Human Cervical Explant Culture. Antimicrob Agents Chemother 51: 1770–1779 doi:10.1128/AAC.01129-06 17353237 10.1128/AAC.01129-06 PMC1855576

[39] Ham AS, Rohan LC, Boczar A, Yang L, W. Buckheit K, et al (2012) Vaginal Film Drug Delivery of the Pyrimidinedione IQP-0528 for the Prevention of HIV Infection. Pharmaceutical Research 29: 1897–1907 doi:10.1007/s11095-012-0715-7 22392331 10.1007/s11095-012-0715-7 PMC4405790

[40] Fichorova RN, Tucker LD, Anderson DJ (2001) The Molecular Basis of Nonoxynol-9-Induced Vaginal Inflammation and Its Possible Relevance to Human Immunodeficiency Virus Type 1 Transmission. Journal of Infectious Diseases 184: 418 –428. doi:10.1086/322047. 10.1086/322047 11471099

[41] Han YA, Singh M, Saxena BB (2007) Development of vaginal rings for sustained release of nonhormonal contraceptives and anti-HIV agents. Contraception 76: 132–138 doi:10.1016/j.contraception.2007.04.006 17656183 10.1016/j.contraception.2007.04.006

[42] Boulmedarat L, Bochot A, Lesieur S, Fattal E (2005) Evaluation of buccal methyl-β-cyclodextrin toxicity on human oral epithelial cell culture model. Journal of Pharmaceutical Sciences 94: 1300–1309 doi:10.1002/jps.20350 15858859 10.1002/jps.20350

[43] Schlievert P, Strandberg K, Brosnahan A, Haase A, Pambuccian S, et al (2008) Glycerol monolaurate does not alter rhesus macaque (Macaca mulatta) vaginal lactobacilli and is safe for chronic use. Antimicrobial Agents and Chemotherapy 52: 4448–4454. 18838587 10.1128/AAC.00989-08 PMC2592867

[44] Lin Y-C, Schlievert PM, Anderson MJ, Fair CL, Schaefers MM, et al (2009) Glycerol Monolaurate and Dodecylglycerol Effects on Staphylococcus aureus and Toxic Shock Syndrome Toxin-1 In Vitro and In Vivo. PLoS ONE 4: e7499 doi:10.1371/journal.pone.0007499 19838303 10.1371/journal.pone.0007499 PMC2759527

[45] Vetter SM, Schlievert PM (2005) Glycerol Monolaurate Inhibits Virulence Factor Production in Bacillus anthracis. Antimicrob Agents Chemother 49: 1302–1305 doi:10.1128/AAC.49.4.1302-1305.2005 15793101 10.1128/AAC.49.4.1302-1305.2005 PMC1068626

[46] Projan SJ, Brown-Skrobot S, Schlievert PM, Vandenesch F, Novick RP (1994) Glycerol Monolaurate Inhibits the Production of Beta-Lactamase, Toxic Shock Toxin-1, and Other Staphylococcal Exoproteins by Interfering with Signal Transduction. J Bacteriol 176: 4204–4209. 8021206 10.1128/jb.176.14.4204-4209.1994 PMC205630

[47] Strandberg KL, Peterson ML, Lin Y-C, Pack MC, Chase DJ, et al (2009) Glycerol Monolaurate Inhibits Candida and Gardnerella vaginalis In Vitro and In Vivo but Not Lactobacillus. Antimicrobial Agents and Chemotherapy 54: 597–601 doi:10.1128/AAC.01151-09 20008774 10.1128/AAC.01151-09 PMC2812150

[48] Watts DH, Rabe L, Krohn MA, Aura J, Hillier SL (1999) The Effects of Three Nonoxynol-9 Preparations on Vaginal Flora and Epithelium. J Infect Dis 180: 426–437 doi:10.1086/314881 10395859 10.1086/314881

[49] Patton DL, Kiddera GG, Sweeneya YC, Rabeb LK, Hillier SL (1999) Effects of multiple applications of benzalkonium chloride and nonoxynol 9 on the vaginal epithelium in the pigtailed macaque (Macaca nemestrina). American Journal of Obstetrics and Gynecology 180: 1080–1087 doi:10.1016/S0002-9378(99)70598-3 10329859 10.1016/s0002-9378(99)70598-3

[50] Moench TR, Mumper RJ, Hoen TE, Sun M, Cone RA (2010) Microbicide excipients can greatly increase susceptibility to genital herpes transmission in the mouse. BMC Infectious Diseases 10: 331 doi:10.1186/1471-2334-10-331 21087496 10.1186/1471-2334-10-331 PMC2996397

[51] Schlievert PM, Deringer JR, Kim MH, Projan SJ, Novick RP (1992) Effect of glycerol monolaurate on bacterial growth and toxin production. Antimicrob Agents Chemother 36: 626–631 doi:10.1128/AAC.36.3.626 1622174 10.1128/aac.36.3.626 PMC190568

[52] Major I, Lowry D, Malcolm K, Woolfson D, Cohen J, et al (2010) Development of a microbicide-releasing diaphragm as an HIV prevention strategy. Conference proceedings: Annual International Conference of the IEEE Engineering in Medicine and Biology Society IEEE Engineering in Medicine and Biology Society Conference 2010: 1089–92. 10.1109/IEMBS.2010.5627333 21096558

[53] Ballagh SA, Brache V, Mauck C, Callahan MM, Cochon L, et al (2008) A phase I study of the functional performance, safety and acceptability of the BufferGel® Duet (TM). Contraception 77: 130–137. 18226678 10.1016/j.contraception.2007.10.003

[54] Schwartz JL, Ballagh SA, Creinin MD, Rountree RW, Kilbourne-Brook M, et al (2008) SILCS diaphragm: postcoital testing of a new single-size contraceptive device. Contraception 78: 237–244 doi:10.1016/j.contraception.2008.04.118 18692615 10.1016/j.contraception.2008.04.118

[55] Free MJ, Srisamang V, Vail J, Mercer D, Kotz R, et al (1996) Latex rubber condoms: predicting and extending shelf life. Contraception 53: 221–229. 8706440 10.1016/0010-7824(96)00041-8

[56] Owen D, Katz D (1999) A vaginal fluid simulant. Contraception 59: 91–5. 10361623 10.1016/s0010-7824(99)00010-4

[57] Notari S, Tommasi C, Nicastri E, Bellagamba R, Tempestilli M, et al (2009) Simultaneous determination of maraviroc and raltegravir in human plasma by HPLC-UV. IUBMB Life 61: 470–470–475. 19319971 10.1002/iub.181

[58] Takeuchi Y, McClure MO, Pizzato M (2008) Identification of Gammaretroviruses Constitutively Released from Cell Lines Used for Human Immunodeficiency Virus Research. J Virol 82: 12585–12588 doi:10.1128/JVI.01726-08 18842727 10.1128/JVI.01726-08 PMC2593302

[59] Derdeyn CA, Decker JM, Sfakianos JN, Wu X, O'Brien WA, et al (2000) Sensitivity of human immunodeficiency virus type 1 to the fusion inhibitor T-20 is modulated by coreceptor specificity defined by the V3 loop of gp120. J Virol 74: 8358–8367. 10954535 10.1128/jvi.74.18.8358-8367.2000 PMC116346

[60] Platt EJ, Wehrly K, Kuhmann SE, Chesebro B, Kabat D (1998) Effects of CCR5 and CD4 Cell Surface Concentrations on Infections by Macrophagetropic Isolates of Human Immunodeficiency Virus Type 1. J Virol 72: 2855–2864. 9525605 10.1128/jvi.72.4.2855-2864.1998 PMC109730

[61] Wei X, Decker JM, Liu H, Zhang Z, Arani RB, et al (2002) Emergence of Resistant Human Immunodeficiency Virus Type 1 in Patients Receiving Fusion Inhibitor (T-20) Monotherapy. Antimicrobial Agents and Chemotherapy 46: 1896–1905 doi:10.1128/AAC.46.6.1896-1905.2002 12019106 10.1128/AAC.46.6.1896-1905.2002 PMC127242

[62] World Health Organization (2010) WHO laboratory manual for the examination and processing of human semen. Geneva: World Health Organization.

