# Supplementary figures and images for: Drug-Eluting Fibers for HIV-1 Inhibition and Contraception
Source: PLoS One. 2012 Nov 28;7(11):e49792. doi: 10.1371/journal.pone.0049792 (PMC3509119; doi:10.1371/journal.pone.0049792)

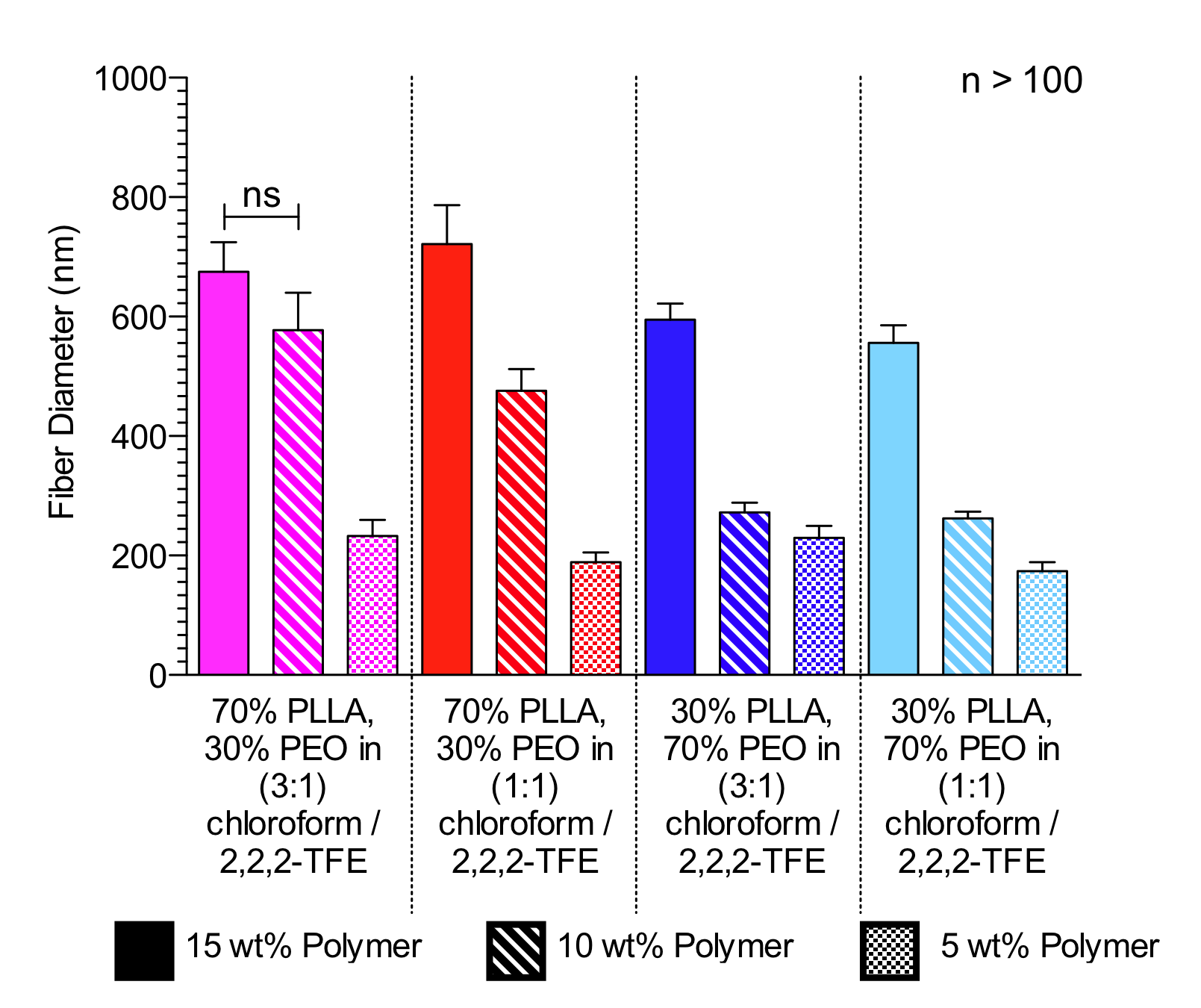

Supplement: Figure S1 — Videos show sperm motility at 5 min after addition of GML, PBS, or Nonoxynol 9. Fiber diameters depend upon polymer viscosity, composition, and solvent. Geometric mean fiber diameters with 95% confidence intervals are shown for 70∶30 PLLA/PEO fibers (red) and 30∶70 PLLA/PEO fibers (blue) made from polymers dissolved in either 1∶1 or 3∶1 (vol/vol) chloroform/2,2,2-trifluoroethanol). Geometric mean fiber diameter was found to vary significantly between all groups based on the concentration of polymer in the electrospinning solution (solid = 15% wt/vol, dashed = 10% wt/vol, dots = 5% wt/vol) (P<0.0001), except as indicated. (TIFF) [file pone.0049792.s001.tif]

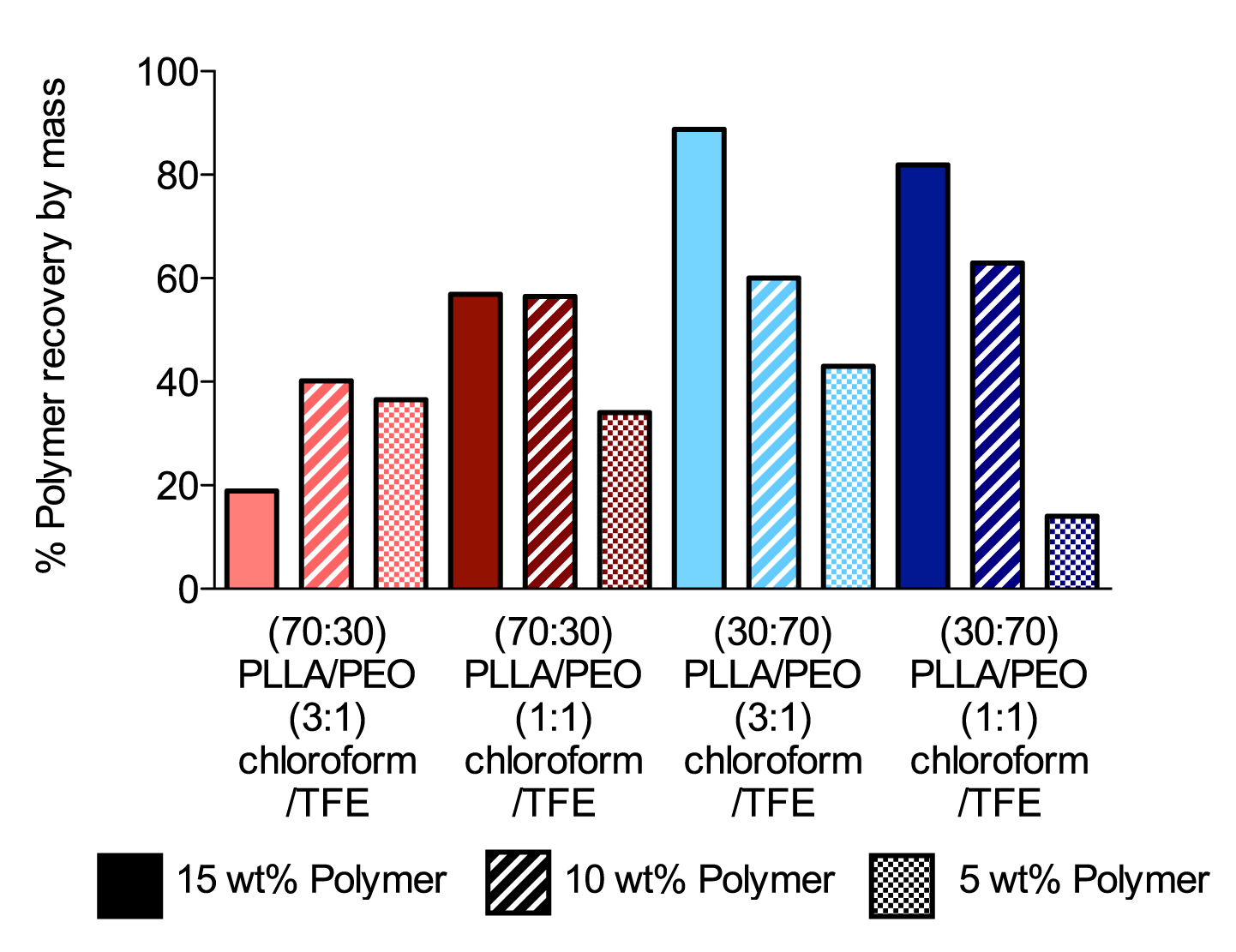

Supplement: Figure S2 — Material efficiency was positively correlated with % wt/vol of polymer in solution for most polymer blends. Here we define material efficiency as the percent polymer recovered from the mandrel by mass. For 30∶70 PLLA/PEO blends, material efficiency increased with the % wt/vol of polymer in solution. Material efficiency is important for cost effectiveness. Based on these results, we chose the 15% (wt/vol) 70∶30 PLLA/PEO in 1∶1 chloroform/TFE and 15% wt/vol 30∶70 PLLA/PEO in 3∶1 chloroform/TFE as the base formulations for all further work with drug encapsulation. (TIF) [file pone.0049792.s002.tif]

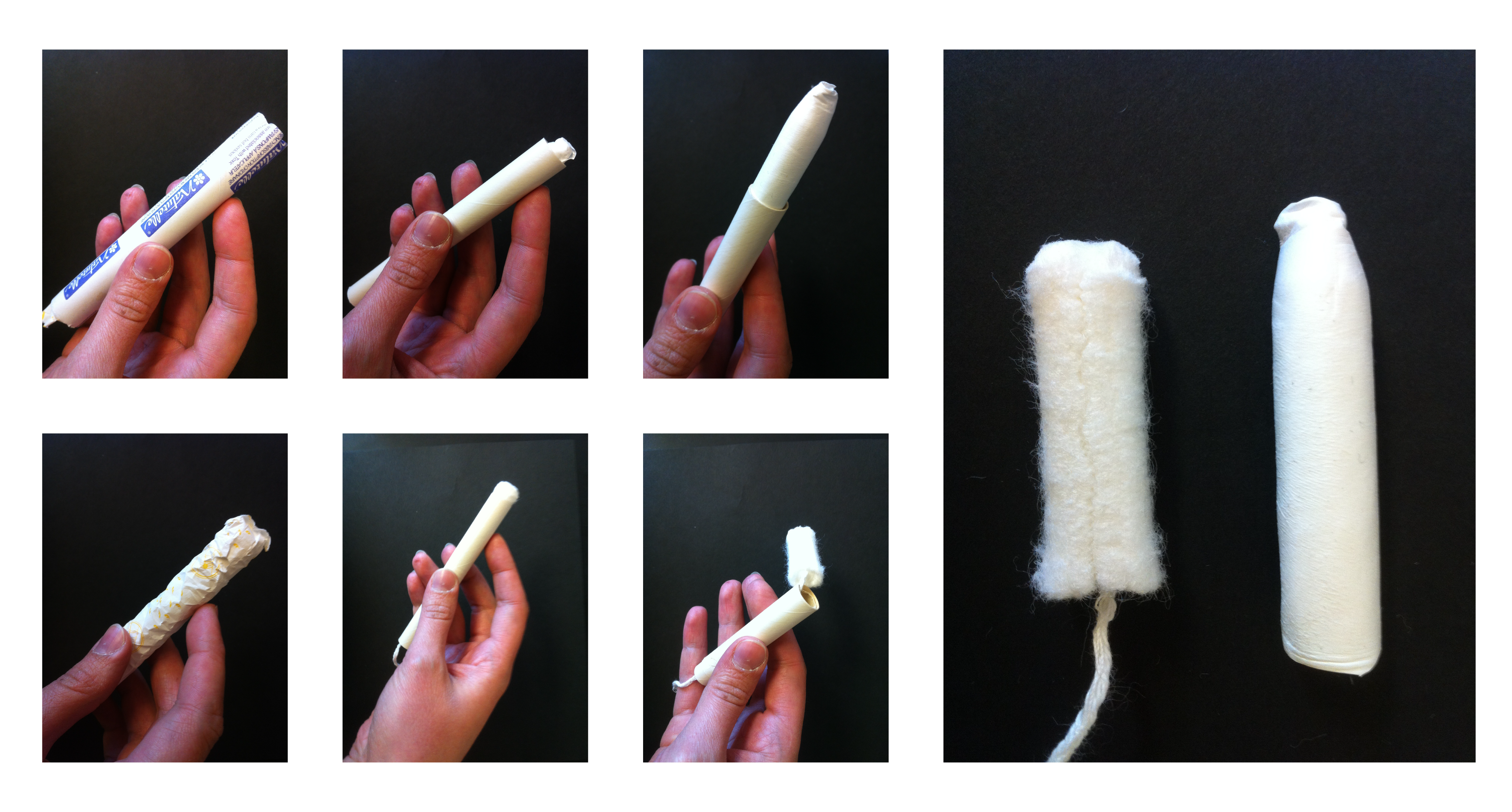

Supplement: Figure S3 — Electrospun fibers can be made sufficiently thick to be easily pushed out of a tampon applicator. The mesh device can be loaded into standard packaging (top and bottom images on the left), and resembles a tampon once opened (2nd to left, top – mesh, bottom – tampon). The device is delivered by pushing the inner cardboard tube through the outer cardboard tube of the applicator, revealing the fiber mesh, which would then hydrate and release active agents (3rd to left, top – mesh, bottom – tampon). A side-by-side comparison of a tampon and the tubular mesh reveals comparable geometry (right). (TIF) [file pone.0049792.s003.tif]

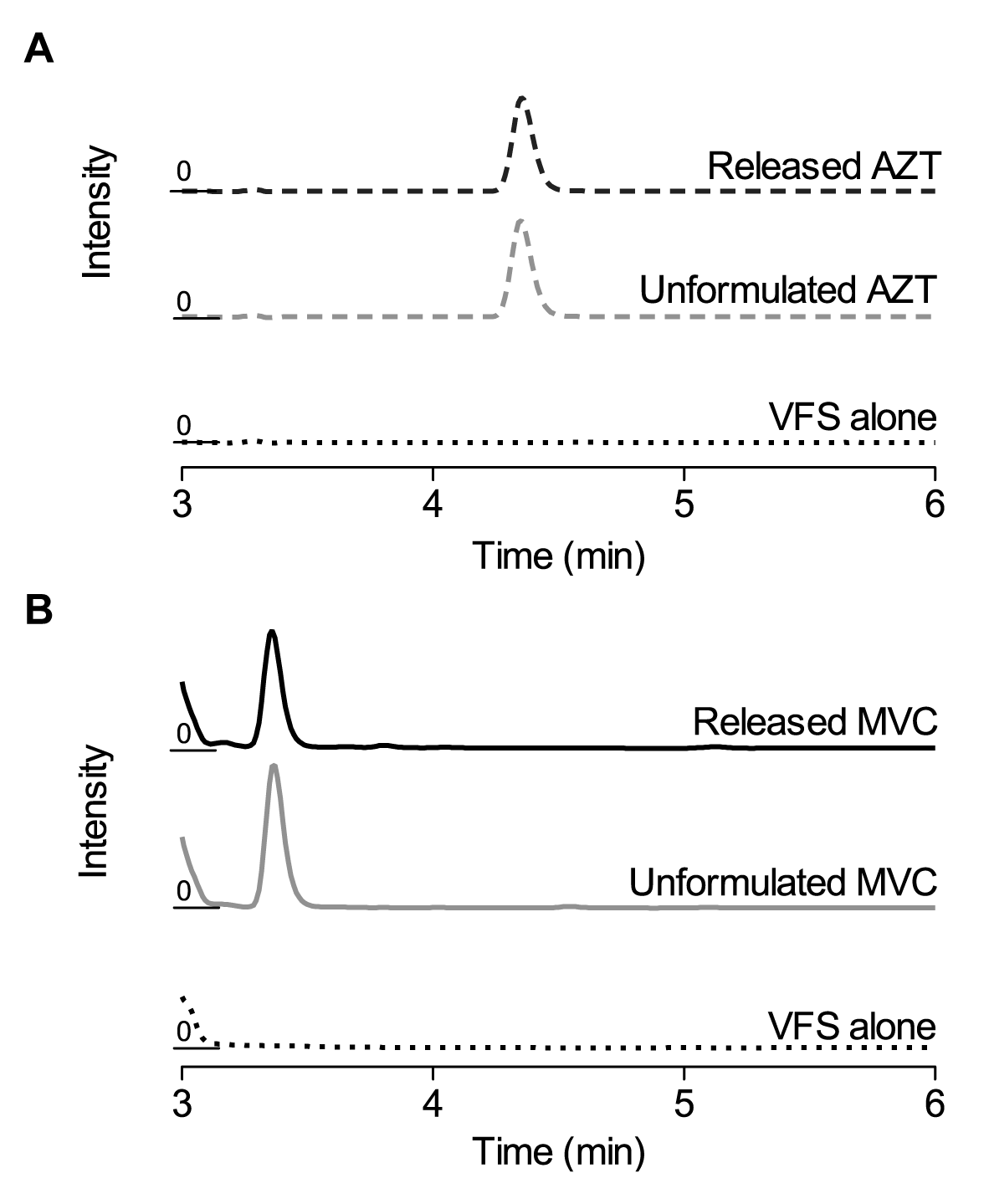

Supplement: Figure S4 — Electrospinning does not alter drug retention time with HPLC. (a) Representative chromatogram for AZT. (b) Representative chromatogram for MVC. Peak shapes and retention times do not change for unformulated drugs (gray lines) compared with drugs released from fibers (black lines). (TIF) [file pone.0049792.s004.tif]

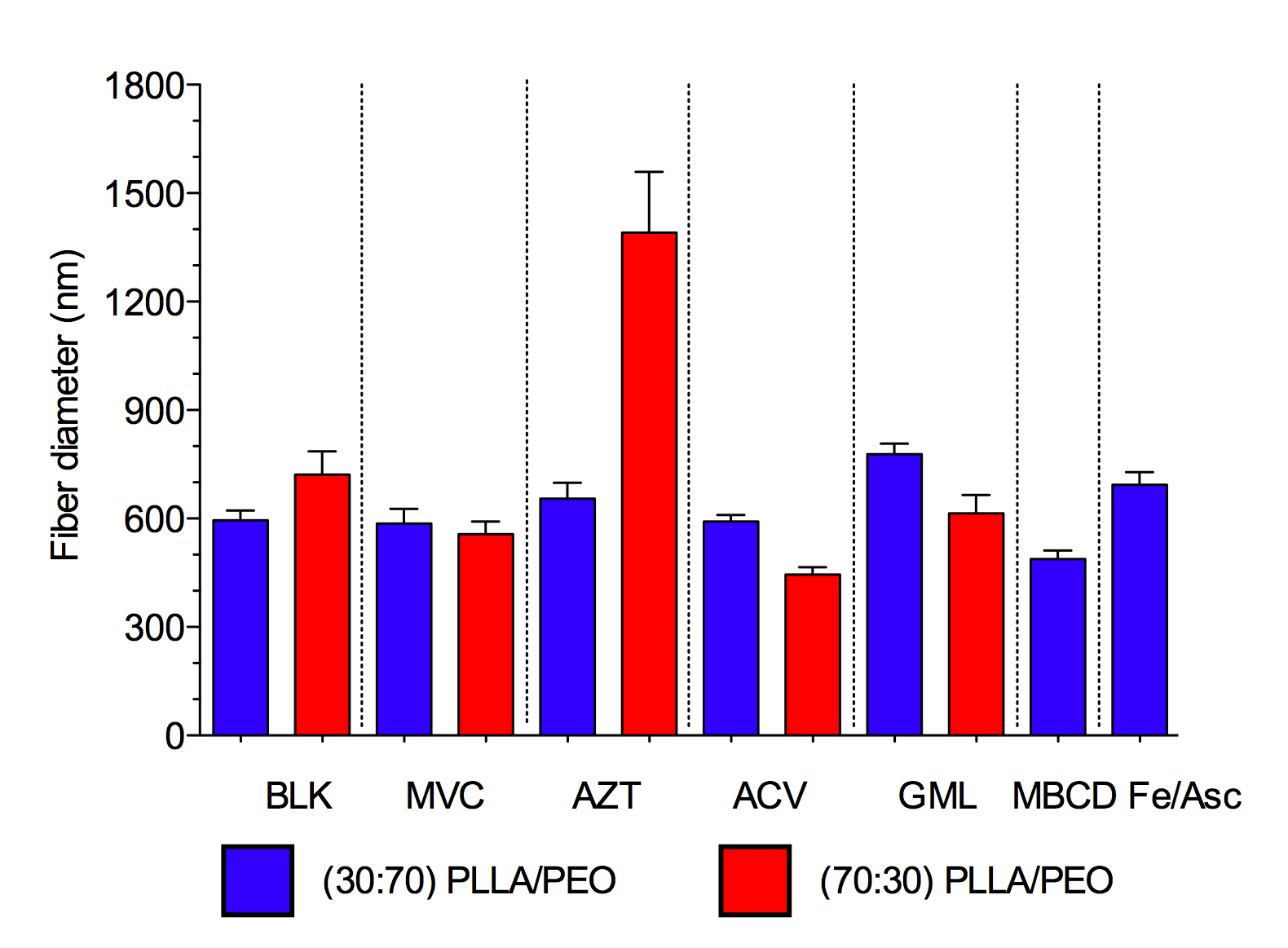

Supplement: Figure S5 — Drug incorporation into 30∶70 and 70∶30 PLLA/PEO electrospun fibers can alter fiber diameter distributions. Geometric mean fiber diameters with 95% confidence intervals are shown for 70∶30 PLLA/PEO fibers (red) and 30∶70 PLLA/PEO fibers (blue) incorporating MVC, AZT, ACV, GML, MBCD, or Fe/Asc compared to blank fibers. (TIFF) [file pone.0049792.s005.tif]

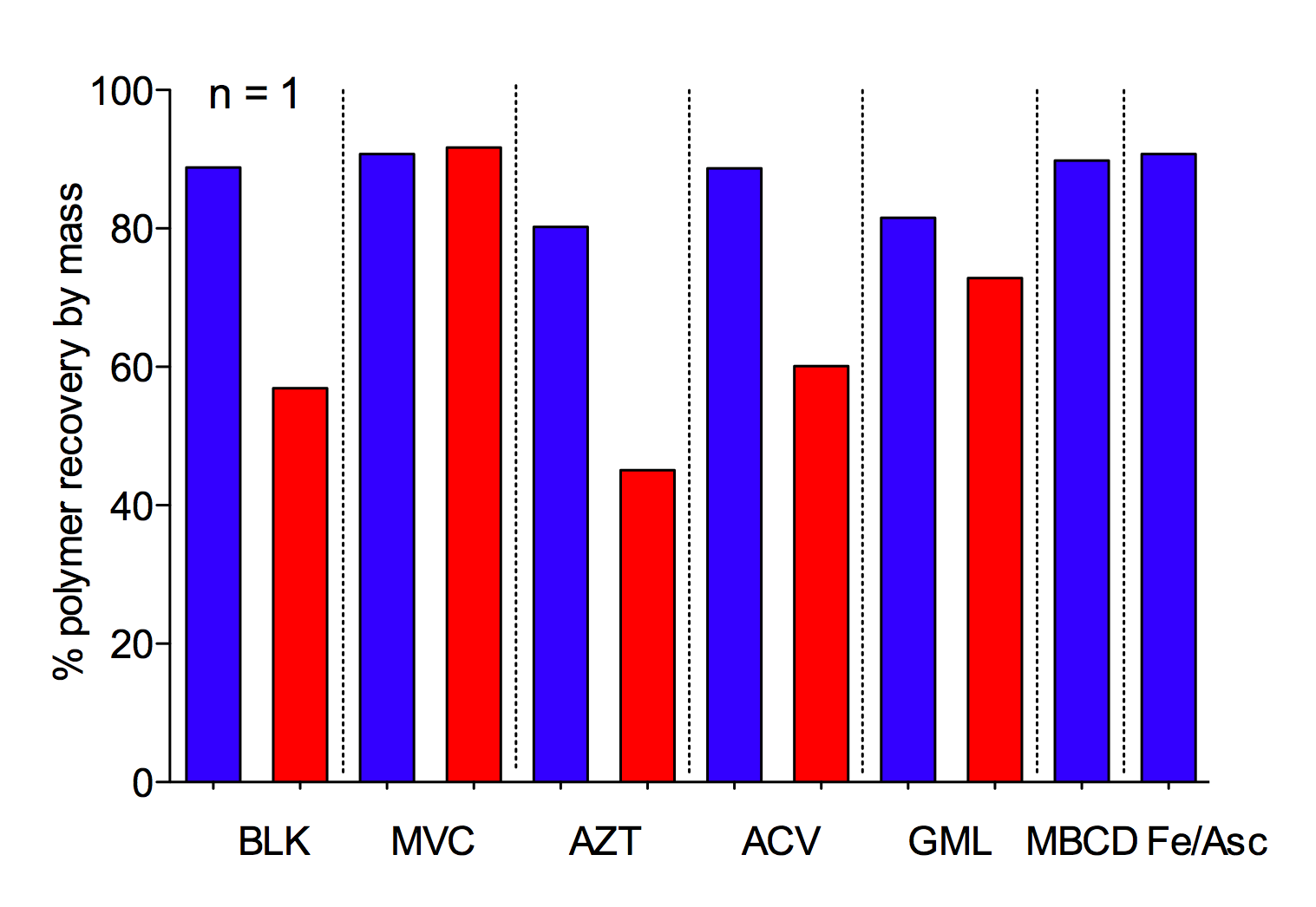

Supplement: Figure S6 — Agent incorporation into fibers influences material efficiency. Incorporating drugs into 30∶70 and 70∶30 PLLA/PEO blends (blue and red, respectively) affected polymer recovery. Interestingly, MVC dramatically increased material efficiency for 70∶30 PLLA/PEO fibers. (TIFF) [file pone.0049792.s006.tif]

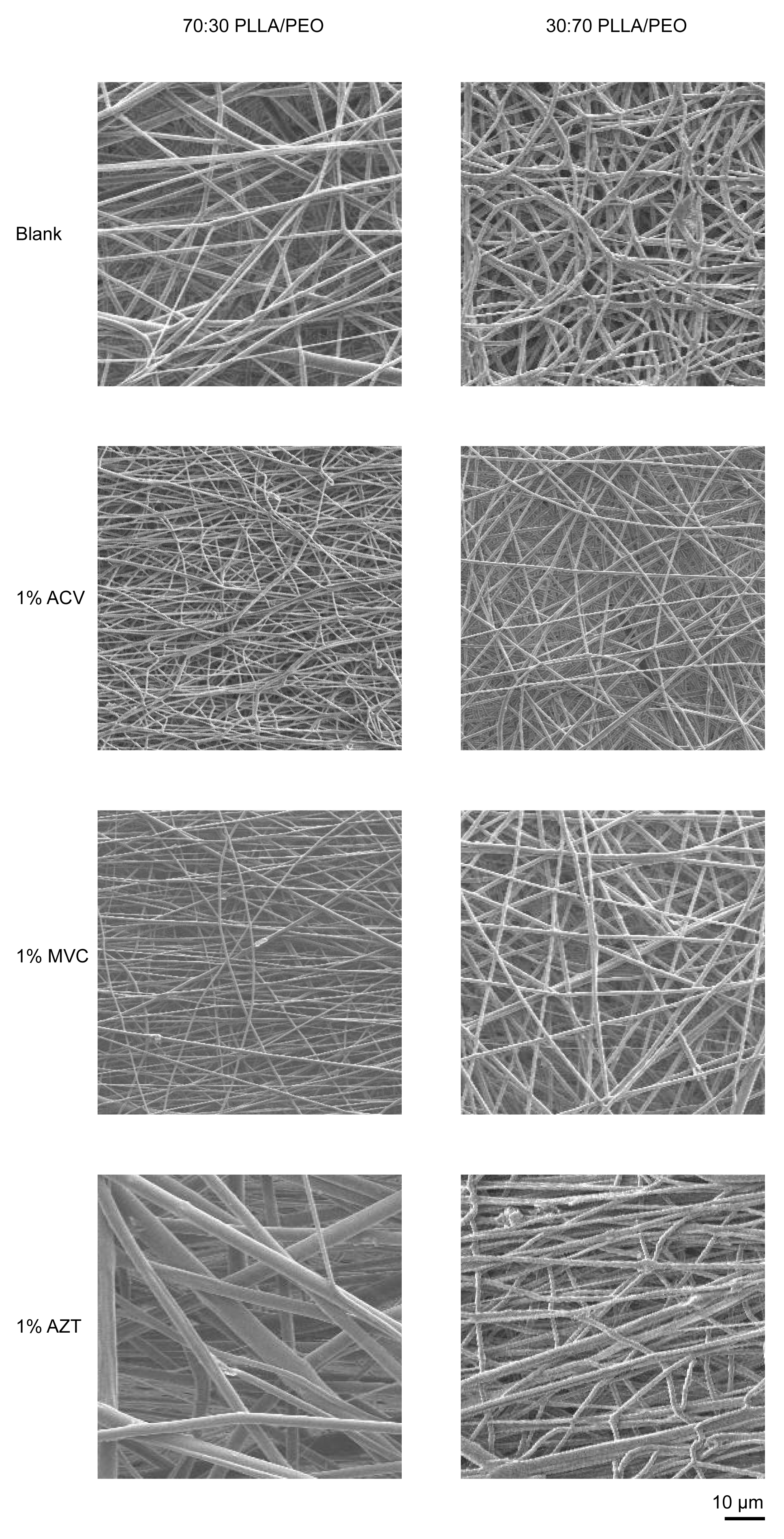

Supplement: Figure S7 — Fiber morphology and alignment for antiviral compounds ACV, MVC, and AZT. Increased alignment of 70∶30 PLLA/PEO fibers is apparent in 1% MVC samples. Increased alignment of 30∶70 PLLA/PEO fibers is apparent in 1% AZT samples. Differences in fiber diameter, previously shown in Fig. S5, are also apparent. (TIF) [file pone.0049792.s007.tif]

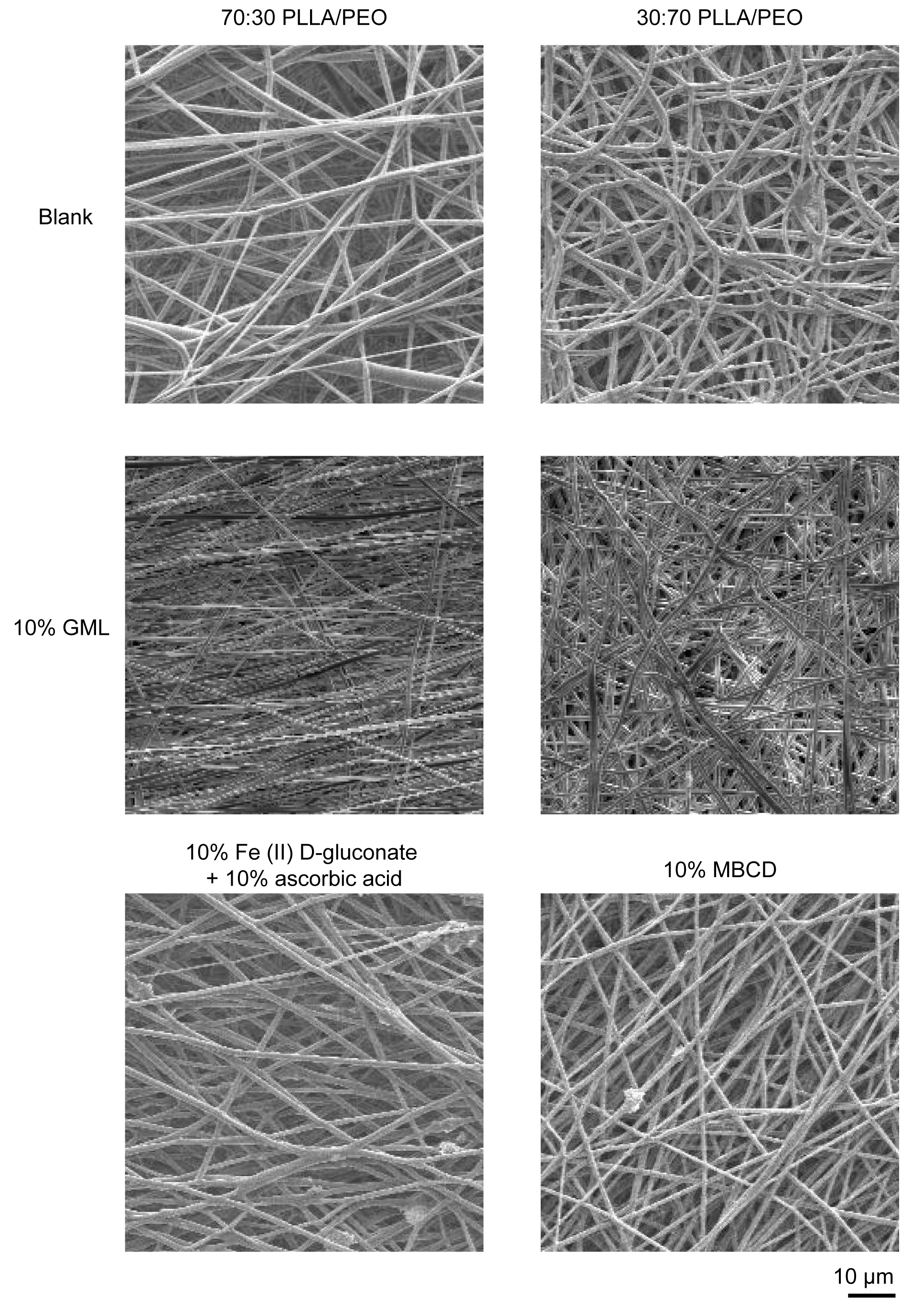

Supplement: Figure S8 — Fiber morphology and alignment for contraceptive compounds GML, Fe/Asc, and MBCD. Increased alignment of 70∶30 PLLA/PEO fibers is apparent in 10% GML samples. Increased alignment of 30∶70 PLLA/PEO fibers is apparent in 10% MBCD samples. Differences in fiber diameter, previously shown in Supporting Fig. S5, are also apparent. (TIF) [file pone.0049792.s008.tif]

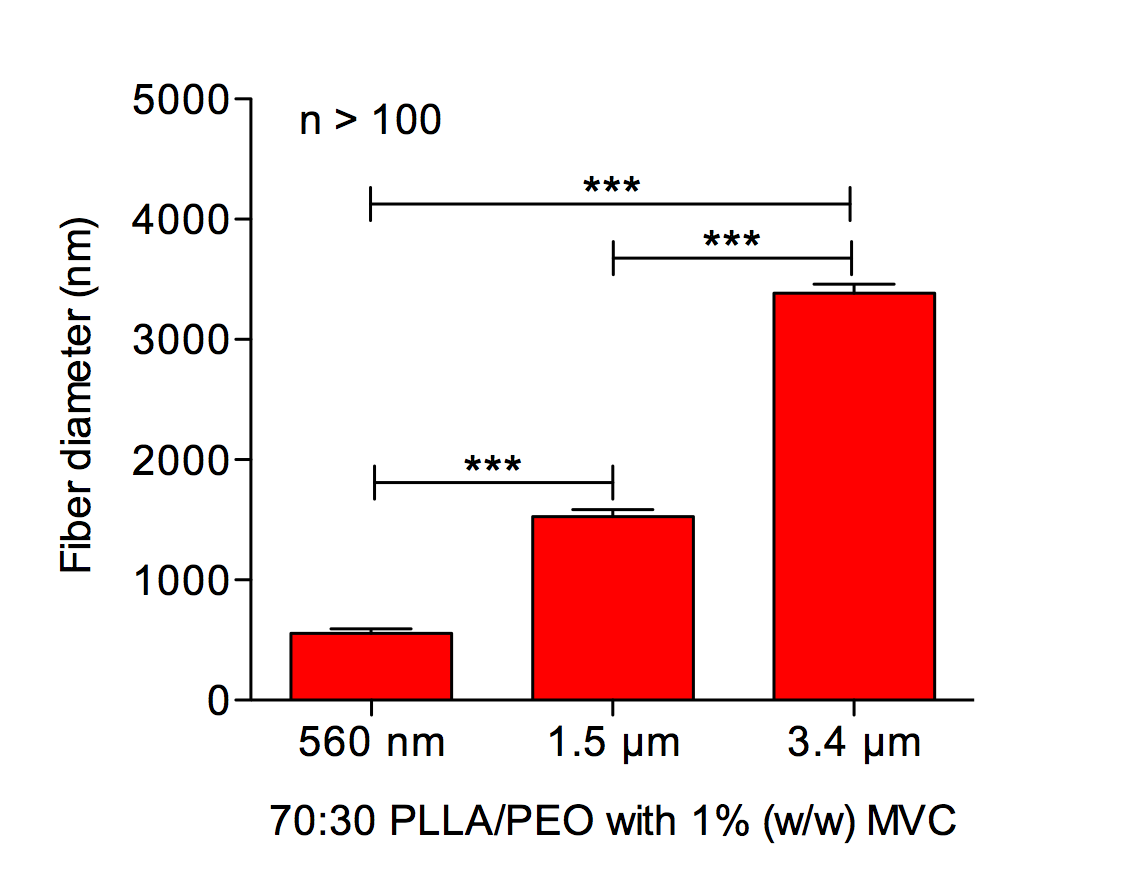

Supplement: Figure S9 — Fiber diameters of 70∶30 PLLA/PEO with 1% (w/w) MVC. Geometric mean fiber diameters with 95% confidence intervals are displayed for 70∶30 PLLA/PEO fibers with 1% (w/w) MVC made by varying polymer concentration and electrospinning parameters. Geometric mean fiber diameters of all three mesh types are significantly different from each other (p<0.0001). These fibers correspond to cumulative release curves displayed in Fig. 3b. (TIFF) [file pone.0049792.s009.tif]

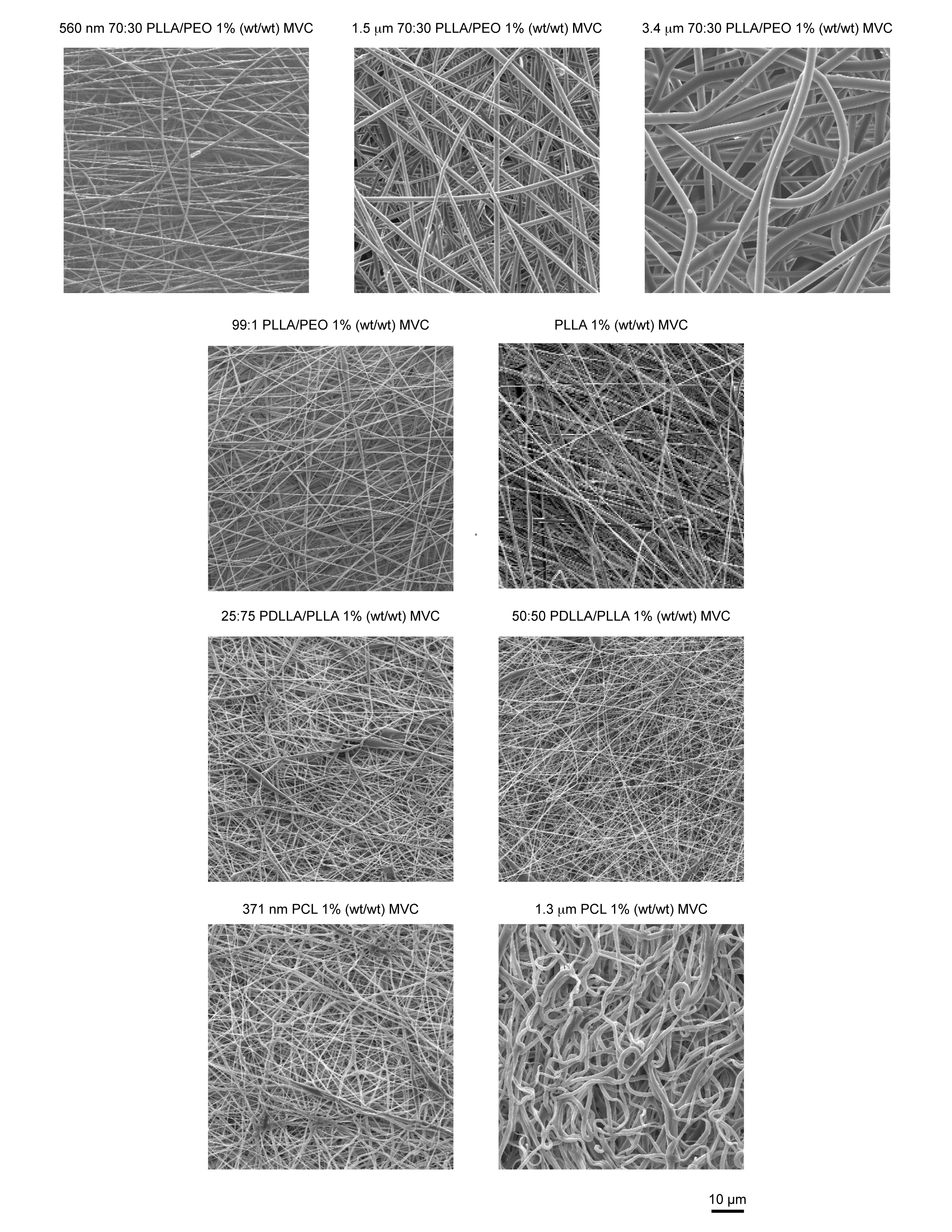

Supplement: Figure S10 — Morphology of additional polymeric fibers incorporating 1% (wt/wt) MVC. Scanning electron micrographs of fibers made from different polymers incorporating 1% (wt/wt) MVC are shown, including 70∶30 PLLA/PEO of three fiber diameters (560 nm, 1.5 μm, and 3.4 μm), 99∶1 PLLA/PEO, PLLA, 25∶75 PDLLA/PLLA, 50∶50 PDLLA/PLLA, and PCL of two fiber diameters (371 nm and 1.3 μm). These fibers correspond to cumulative release curves displayed in Fig. 3. (TIF) [file pone.0049792.s010.tif]

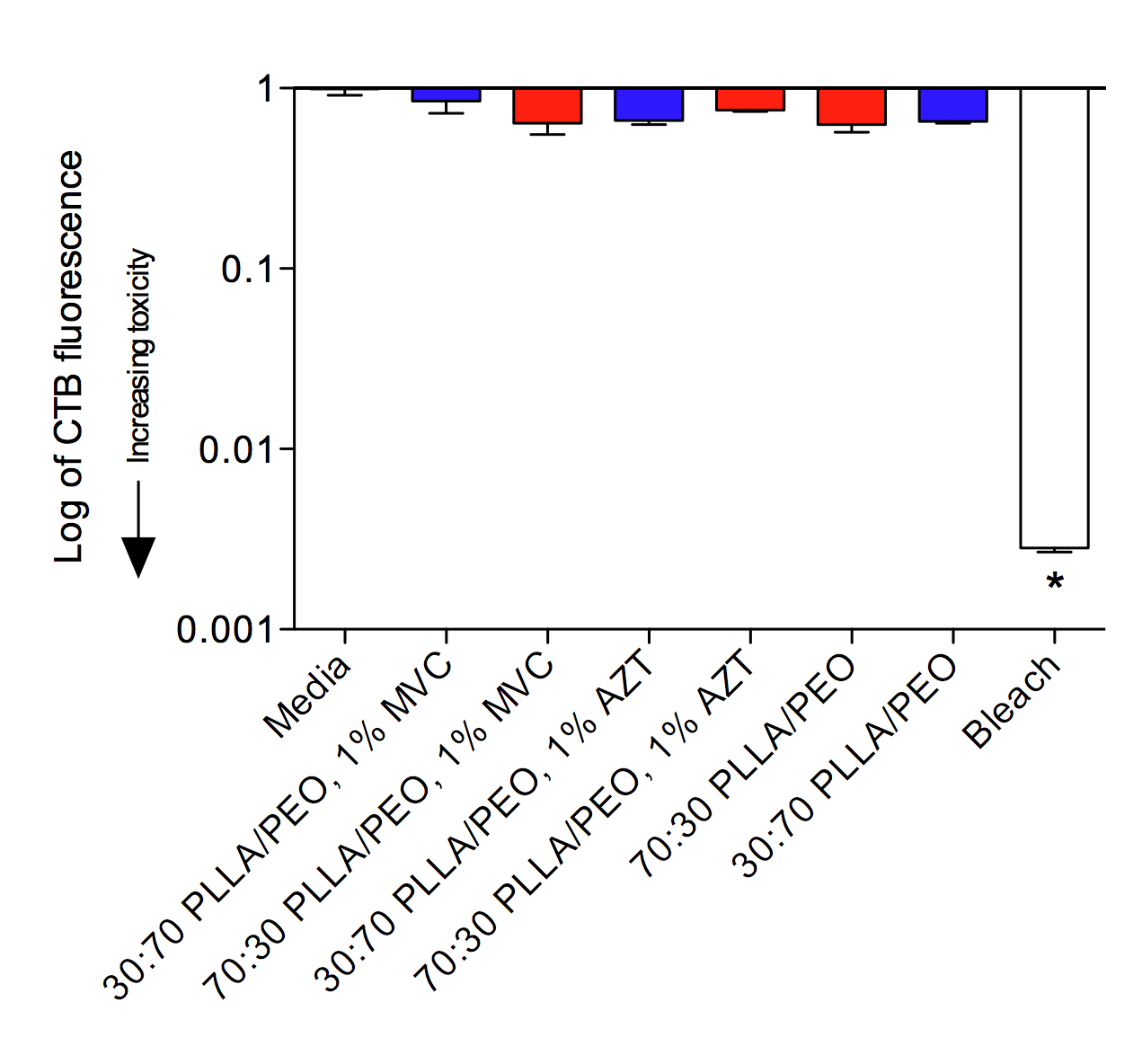

Supplement: Figure S11 — Fibers are not cytotoxic to TZM-bl cells. Cytotoxicity was tested using the CellTiter-BlueTM cell viability assay by culturing TZM-bL cells with disks of blank and drug-loaded meshes (n = 3) for 48 h. Fluorescence signals for each condition are plotted as log-transformed values. Media-only treated cells and fiber-treated cells show significantly less cytotoxicity than bleach-treated cells (p<0.05). (TIFF) [file pone.0049792.s011.tif]

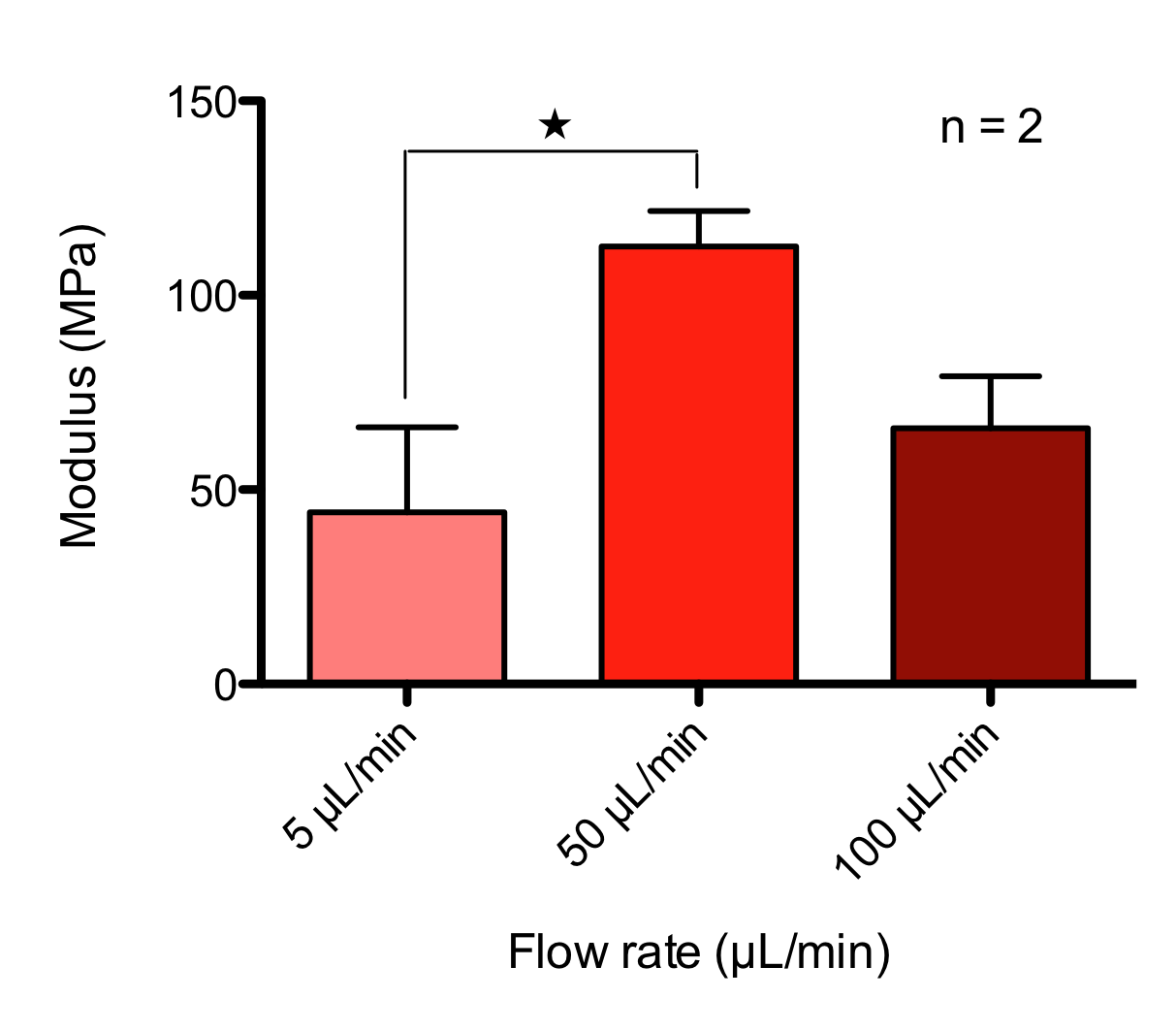

Supplement: Figure S12 — Flow rate influences modulus of PCL fibers incorporating 1% (w/w) MVC. Moduli of PCL fiber meshes made from electrospinning at varying flow rates (5, 50, and 100 μL/min) were measured. The modulus of PCL fibers electospun at a flow rate of 50 μl/min is significantly different than the modulus of PCL fibers electrospun at 5 μL/min (p<0.05). (TIFF) [file pone.0049792.s012.tif]
