# Supplementary material for: Drug-Eluting Fibers for HIV-1 Inhibition and Contraception
Source: PLoS One. 2012 Nov 28;7(11):e49792. doi: 10.1371/journal.pone.0049792 (PMC3509119; doi:10.1371/journal.pone.0049792)
Supplement: Table S1 — GML inhibits sperm motility in swimout sperm at 0.05 and 0.5% (wt/vol). We tested the contraceptive agents for their ability to inhibit sperm motility in a dose response motility experiment. Equal amounts of drug solution and human swimout sperm were combined on a glass slide. Changes in sperm motility over 2–5 min were recorded using a microscope and video recording system. Time point controls with PBS were performed to ensure drug effects on motility were independent of sperm incubation time. PBS percent motility at 2 min reflects average over all PBS control measurements, with range from 72%–100%. (DOC) [file pone.0049792.s017.doc]

**Table S1—GML inhibits sperm motility in swimout sperm at 0.05 and 0.5% (wt/vol).**

| **Solution** | **Concentration (wt/vol)** | **Percent Motility at 2 min** | **Complete Inhibition within 5 min** |
| --- | --- | --- | --- |
| PBS | 1x | 89 | No |
| Nonoxynol-9 | 0.4% | 0 | Yes (< 30 s) |
| Glycerol Monolaurate | 0.5% (pH 7) | 30 | Yes (< 4 min) |
|  | 0.05% | 28 | Yes (< 5 min) |
|  | 0.005% | 55 | No |
|  | 0.0005% | 84 | No |
|  | 0.00005% | 75 | No |
| Fe(II) D-gluconate | 5% | 52 | No |
| L-ascorbic acid | 5% (pH 2) | 0 | Yes (< 30 s) |
|  | 0.5% (pH 4) | 21 | No |
| Fe(II) D-gluconate + L-ascorbic acid | 5% / 5% | 0 | Yes (< 30 s) |
|  | 0.5% / 0.5% (pH 4) | 12 | No |
|  | 0.05% / 0.05% (pH 5.5) | 85 | No |
| Methyl-β-cylcodextrin | 5% | 94 | No |

We tested the contraceptive agents for their ability to inhibit sperm motility in a dose response motility experiment. Equal amounts of drug solution and human swimout sperm were combined on a glass slide. Changes in sperm motility over 2–5 min were recorded using a microscope and video recording system. Time point controls with PBS were performed to ensure drug effects on motility were independent of sperm incubation time. PBS percent motility at 2 min reflects average over all PBS control measurements, with range from 72%–100%.
