# Supplementary material for: Drug-Eluting Fibers for HIV-1 Inhibition and Contraception
Source: PLoS One. 2012 Nov 28;7(11):e49792. doi: 10.1371/journal.pone.0049792 (PMC3509119; doi:10.1371/journal.pone.0049792)
Supplement: Table S2 — Electrospun fibers effectively block sperm migration in a transwell assay. We tested the ability of electrospun fiber mats to block sperm migration in a transwell assay. 30∶70 PLLA/PEO fibers without drug were used to replace existing membranes in transwell cups. Motile sperm collected by swimout into media were added to the upper chamber (inside of the cup) and allowed to attempt to enter the bottom chamber for 2 hours. Sperm were then counted in both chambers to assess if sperm could penetrate the meshes. (DOC) [file pone.0049792.s018.doc]

**Table S2—Electrospun fibers effectively block sperm migration in a transwell assay.**

|  | **30:70 PLLA/PEO fibers** | **Control membranes** |
| --- | --- | --- |
| Upper chamber | 10 ^6.05 ± 0.036^ sperm mL^-1^ | 10 ^6.52 ± 0.25^ sperm mL^-1^ |
| Bottom chamber | 0 sperm mL^-1^ | 10 ^4.76 ± 0.405^ sperm mL^-1^ |

We tested the ability of electrospun fiber mats to block sperm migration in a transwell assay. 30:70 PLLA/PEO fibers without drug were used to replace existing membranes in transwell cups. Motile sperm collected by swimout into media were added to the upper chamber (inside of the cup) and allowed to attempt to enter the bottom chamber for 2 hours. Sperm were then counted in both chambers to assess if sperm could penetrate the meshes.
